# Raman and surface-enhanced Raman spectroscopy for intraoperative cancer diagnostics: sentinel lymph node biopsy, biomarkers and translational challenges

**DOI:** 10.3389/fbioe.2026.1901620

**Published:** 2026-08-28

**Authors:** Jiaqi Feng, Chunyan Li, Xiaowei Jiang, Lizhi Zhou, Yifei Jiang, Binge Deng

**Affiliations:** 1 Hunan Institute of Advanced Sensing and Information Technology, Xiangtan University, Xiangtan, Hunan, China; 2 Experimental Teaching Center of Biology and Basic Medicine, North Henan Medical University, Xinxiang, Henan, China; 3 Department of Infectious Diseases, Xiangtan Central Hospital, Xiangtan, Hunan, China; 4 Department of Nuclear Medicine and Institute for Medical Imaging Technology, Ruijin Hospital, Shanghai Jiao Tong University School of Medicine, Shanghai, China

**Keywords:** biochemical fingerprint, intraoperative diagnosis, Raman spectroscopy, sentinel lymph node biopsy, surface-enhanced Raman spectroscopy

## Abstract

Sentinel lymph node biopsy (SLNB) serves as the gold standard for staging regional lymph node metastasis of solid tumors, which is crucial for guiding clinical treatment decisions and evaluating prognosis. Conventional intraoperative detection methods for SLN, such as frozen section and touch imprint cytology, have limitations including low sensitivity for micrometastasis, long detection time, and high subjectivity. Raman spectroscopy (RS), as a label-free, non-destructive optical molecular imaging technology, can obtain the biochemical fingerprint information of tissues by detecting the inelastic scattering of photons with biomolecules. A series of derivative techniques, exemplified by Surface-Enhanced Raman Spectroscopy (SERS), further overcome the shortcomings of limited penetration depth and weak inherent signals of RS, realizing ultra-sensitive and targeted detection of SLN. Crucially, the integration of advanced machine learning algorithms and deep learning workflows effectively addresses the multivariate complexity of high-dimensional Raman data, enabling rapid, objective, and automated screening for lymph node metastases. This review systematically summarizes the application progress of RS, SERS and derivative technologies in SLNB of various tumors (including breast cancer, thyroid cancer, melanoma, *etc.*), elaborates their technical principles, application forms (label-free and labeled detection), advantages and limitations in clinical practice, and discusses the current challenges and future development directions of RS technology in the clinical translation of SLNB, aiming to provide insights into the development of rapid, accurate and objective intraoperative SLN detection technology.

## 1 Introduction

### 1.1 Clinical significance of sentinel lymph node biopsy

The SLN is defined as the first lymph node (LN) to receive lymphatic drainage from a primary solid tumor, representing the primary gateway and the most critical predictor of regional tumor dissemination (Nathanson, 2010; Heerdt, 2018). Based on the theory of the orderly progression of lymphatic metastasis, the status of the SLN accurately reflects overall regional involvement, thereby determining whether a patient can safely forgo complete lymph node dissection (CLND) (Nieweg et al., 1999; Veronesi et al., 1998). For patients with clinically node-negative solid tumors such as breast cancer (Heerdt, 2018), melanoma (Faries, 2024), and endometrial cancer (Hanna et al., 2017), accurate identification and pathological assessment of the SLN can significantly reduce overtreatment associated with routine complete axillary or regional LN dissection. Consequently, SLNB has evolved into the standard of care for evaluating regional metastasis in early-stage disease (Cox et al., 1998; Smith et al., 2017; Ogasawara et al., 2008; Kr et al., 2019).

**SCHEME 1:**
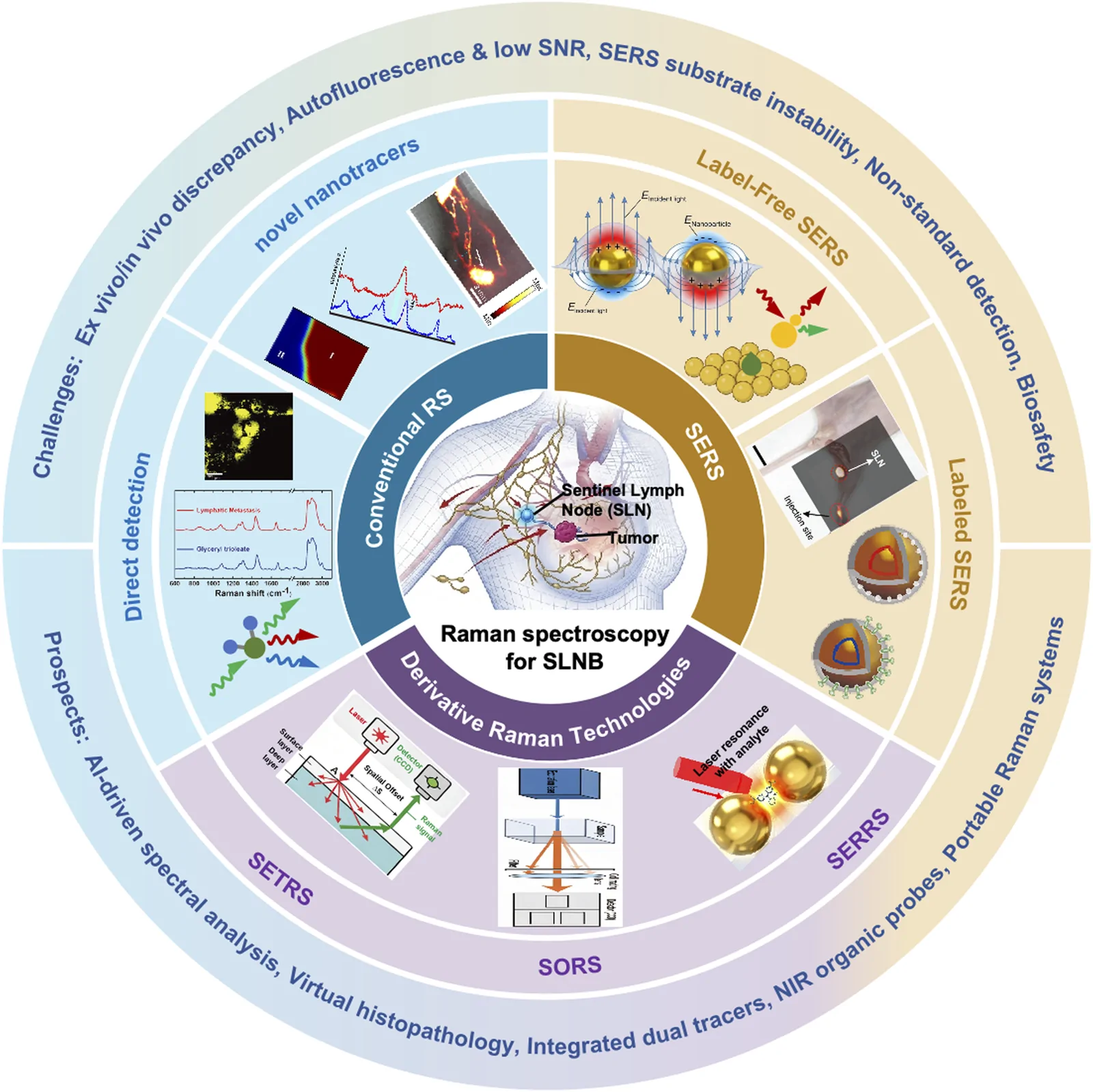
Overview of Raman spectroscopy-guided sentinel lymph node biopsy. “Label-Free SERS”: Original images; “Labeled SERS”: adapted with permission from Zhang et al. 2019, licensed under CC BY 4.0, and Bao et al., 2021, © 2021 Elsevier Ltd, with permission from Elsevier; “SETRS, SORS, SERRS”: Original images; “Direct detection”: adapted with permission from Surmacki et al., 2013, licensed under CC BY 2.0, Zeng et al., 2024, Copyright 2024 American Chemical Society, and original images; “Novel nanotracers”: adapted with permission from Bao et al., 2024, Copyright 2024 American Chemical Society, and Gao et al., 2025, licensed under CC BY 4.0.

**FIGURE 1:**
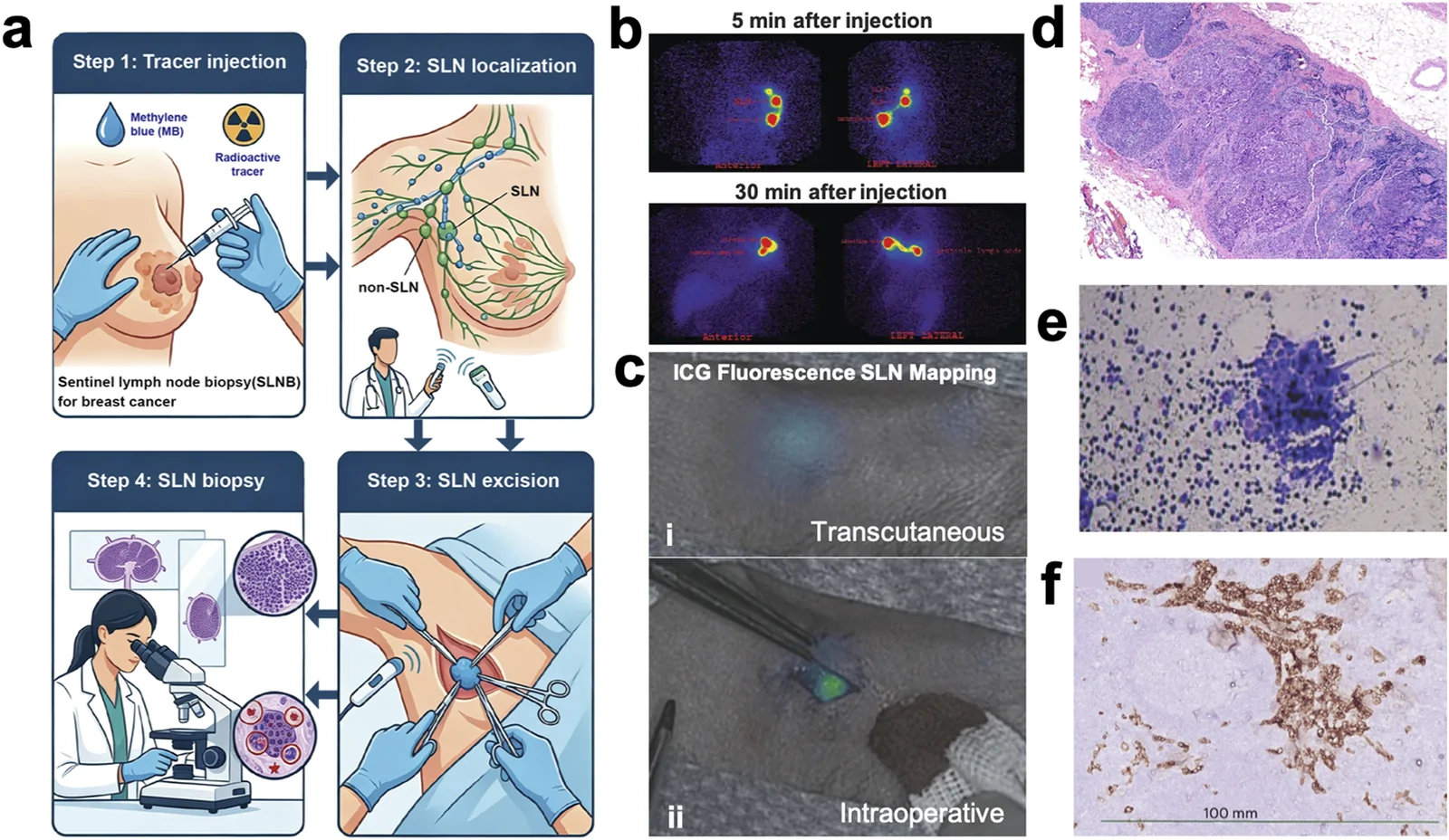
SLNB workflow and intraoperative pathological detection methods of metastatic LNs. **(a)** Schematic diagram of sentinel lymph node biopsy (SLNB) workflow: Injection of tracer (blue dye) around the tumor (Step 1); lymphatic drainage to the sentinel lymph node for localization (Step 2); surgical resection of the SLN (Step 3) for pathological evaluation to guide treatment decisions (Step 4) (Note: Original images). **(b)** Representative lymphoscintigraphy images of axillary SLNs after injection of radioactive tracers at different time points (adapted from Moslehi et al. 2015, licensed under CC BY-NC-SA 3.0). **(c)** Near-infrared (NIR) fluorescence imaging for SLN mapping using indocyanine green (ICG). (i) Transcutaneous fluorescent signal detection. (ii) Intraoperative fluorescent signal detection (reproduced from Zweedijk et al., 2024, licensed under CC BY 4.0). **(d)** Intraoperative HE-stained frozen section of a metastatic sentinel lymph node (adapted from Rau et al. 2022, licensed under CC BY 4.0). **(e)** Touch imprint cytology of a breast cancer sentinel lymph node. Malignant cellular smears showing atypical cell clusters characterized by enlarged, hyperchromatic, and overlapping nuclei on a lymphoid background (adapted from Yadav et al. 2024, licensed under CC BY 4.0). **(f)** IHC staining of a metastatic SLN, highlighting tumor cells to detect micrometastases that may be missed on routine H&E staining (adapted from Van Dooijeweert et al., 2024, licensed under CC BY 4.0).

The workflow of SLNB is illustrated in Figure 1a. The standard SLNB procedure involves injecting a tracing agent near the primary tumor, which migrates through the lymphatic channels to the SLNs (Heerdt, 2018). Surgeons then locate and remove these SLNs based on the signal from the tracer, including blue dye visualization (Figure 1a) (Heerdt, 2018), radioactive detection (Figure 1b) (Ogasawara et al., 2008; Marits et al., 2006; Onof et al., 2024), fluorescence imaging (Figure 1c) (Carlos et al., 2025; Noh et al., 2012; Zweedijk et al., 2024), or magnetic sensing (Voelker and Rebecca, 2018; Finas et al., 2015). The harvested SLNs are subsequently examined pathologically to evaluate metastatic status, guiding subsequent treatment decisions (Heerdt, 2018; Marits et al., 2006).

In breast cancer, SLNB has revolutionized the clinical management of axillary LN status. Traditional axillary lymph node dissection (ALND) carries substantial morbidity and shoulder dysfunction. By enabling accurate intraoperative assessment of LN status, SLNB allows patients with negative SLNs to avoid unnecessary ALND while maintaining staging accuracy (Veronesi et al., 1998; Cox et al., 1998). In Veronesi’s pivotal study, lymphoscintigraphy and γ-probe guidance achieved a 98% identification rate and a low 2.5% false-negative rate, suggesting that this technique may provide equivalent staging information with reduced patient morbidity compared to routine ALND (Veronesi et al., 1998). Cox et al. further validated the combined use of blue dye and radiolabeled colloids, facilitating a paradigm shift toward risk-tailored nodal management, even in low-risk subgroups such as ductal carcinoma *in situ* (DCIS) (Cox et al., 1998). In addition, the pathological information of SLN can also provide important prognostic value for breast cancer patients: the number of metastatic SLNs, the size of metastatic foci, and the presence of extracapsular extension are all independent risk factors for breast cancer recurrence and distant metastasis (Nieweg et al., 1999; Naidoo and Pinder, 2016; Neri et al., 2005).

The clinical utility of SLNB extends to other solid tumors. For melanoma, a highly malignant skin tumor, the SLN represents the primary route of early regional metastasis. In patients with primary melanoma thicker than 0.75 mm, SLNB not only accurately identifies regional LN status but also guides subsequent therapeutic interventions (such as completion lymph node dissection), thereby improving clinical outcomes, particularly in those with intermediate-thickness melanoma (Starz et al., 2004). In endometrial cancer, SLNB is a highly accurate modality for LN staging (sensitivity 96%, negative predictive value 99.7%) and a promising alternative standard of care in early-stage patients (Smith et al., 2017). For thyroid cancer, especially clinically node-negative (cN0) papillary thyroid cancer with a high rate of occult cervical LNM, SLNB can effectively locate metastatic cervical SLNs, enabling precise LN dissection and minimizing potential damage to the parathyroid glands and recurrent laryngeal nerve associated with extensive cervical LN dissection (Garau et al., 2019).

Despite the significant clinical benefits of SLNB, the accuracy of intraoperative SLN metastasis detection is the key to its clinical application, and the traditional intraoperative pathological detection methods have obvious limitations and clinical challenges, which restrict the further optimization of SLNB clinical efficacy. At present, the main intraoperative detection methods for SLN include frozen section (FS) examination (Figure 1d) (Moatasi et al., 2013; Vrande et al., 2009; Tanis, 2001), touch imprint cytology (TIC) (Figure 1e) (Chicken et al., 2006), and rapid immunohistochemistry (IHC) (Figure 1f) (Van Dooijeweert et al., 2024; Giobuin et al., 2009), all of which have inherent defects in sensitivity, speed, and objectivity (Vrande et al., 2009; Barkur et al., 2022; Ji et al., 2026). For instance, FS and TIC often struggle to detect micrometastases (<2 mm), with FS being particularly prone to tissue distortion and artifacts (Yan et al., 2025). Furthermore, the turnaround time for these procedures (typically 20–60 min) can prolong anesthesia and increase the risk of postoperative complications (Smith et al., 2003; Papadoliopoulou et al., 2023). These diagnostic gaps create risks of undertreatment from missed metastases or prolonged operative stress. Thus, there is an urgent demand for a rapid (10–20 min), objective, and non-destructive intraoperative technology. As an emerging optical biopsy tool, RS offers a promising solution by providing chemically specific “fingerprints” of nodal tissues, potentially addressing the shortcomings of traditional morphology-based diagnostics (Kong et al., 2015; Auner et al., 2018).

### 1.2 Basic principles and tumor diagnosis of Raman spectroscopy

**FIGURE 2:**
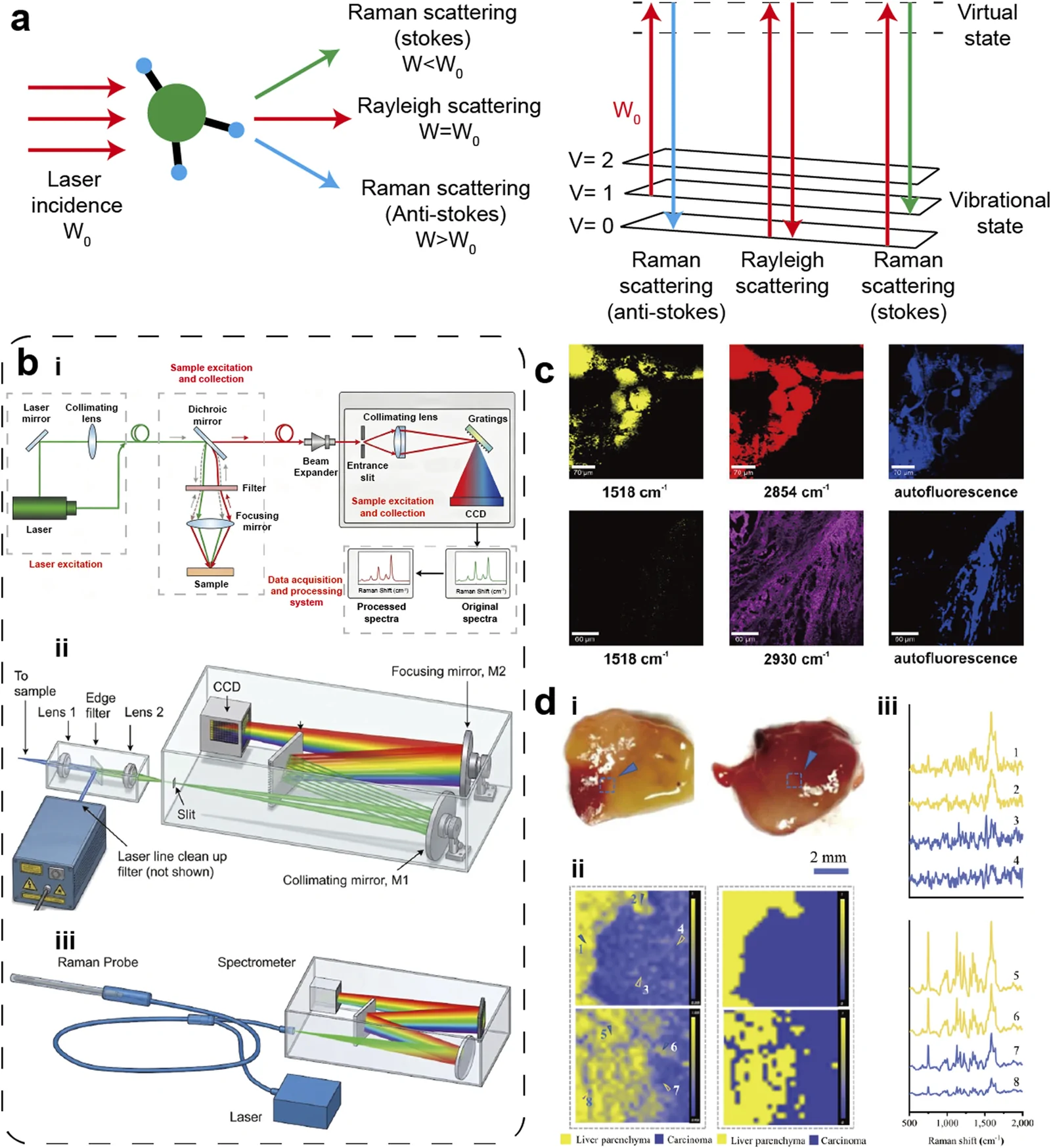
Schematic diagrams of core principles, instrumentation, and tumor diagnosis of RS. **(a)** Schematic illustration and Jablonski energy-level diagram of Raman scattering (Note: Original image). **(b)** A schematic diagram of the optical setup of a Raman spectrometer and Raman spectrometers used for different applications: i) Optical setup of a Raman spectrometer (Note: Original image). ii) Schematic of a laboratory Raman spectrometer: Laser light is focused onto the sample, scattered light is filtered to block Rayleigh scattering, and Raman signals are dispersed by a grating and detected by a CCD (adapted from Auner et al., 2018, licensed under CC BY 4.0). iii) Fiber-optic Raman probe for intraoperative use: Integrates cleanup/edge filters and lenses to transmit laser and collect Raman signals via optical fibers, enabling minimally invasive tissue detection (adapted from Auner et al., 2018, licensed under CC BY 4.0). **(c)** Raman imaging of noncancerous (top) and cancerous (bottom) breast tissue, mapping the spatial distribution of carotenoids (1518 cm^−1^), monounsaturated fatty acids (2854/2930 cm^−1^), and autofluorescence (adapted from Surmacki et al., 2013, licensed under CC BY 2.0). **(d)** Raman spectroscopic identification of tumor margins in liver cancer tissues: (i) Liver cancer blocks with imaging regions (arrows); (ii) Raman images revealing clear tumor margins; (iii) Representative spectra from non-tumor (points 1, 2, 5, 6) and tumor (points 3, 4, 7, 8) regions, showing distinct biochemical fingerprints (adapted from Huang et al., 2023, licensed under CC BY 4.0).

Raman spectroscopy (RS) is a powerful vibrational spectroscopic technique rooted in the Raman scattering effect, a physical phenomenon first discovered by Indian physicist C.V. Raman and his collaborator K.S. Krishnan in 1928 (Raman and Krishnan, 1928). Unlike elastic light scattering (e.g., Rayleigh scattering) where scattered photons retain the same energy and wavelength as incident photons, Raman scattering is an inelastic light-matter interaction, as shown in Figure 2a: when monochromatic laser photons irradiate biological samples, they collide with the chemical bonds of biomolecules (nucleic acids, proteins, lipids, carbohydrates, *etc.*), transferring partial energy to the molecules or absorbing energy from them (Papadoliopoulou et al., 2023; Kong et al., 2015). This energy exchange causes a measurable wavelength shift (Raman shift) of the scattered photons, with the shift value (expressed in wavenumbers, cm^−1^) corresponding to the characteristic vibrational and rotational frequencies of specific chemical bonds in the molecules, and the intensity of the scattered Raman signal reflecting the relative content of the corresponding biomolecules (Diem et al., 2013; Butler et al., 2016; Deng et al., 2024a; Fang et al., 2024).

The typical optical configuration and instruments of a Raman spectrometer are illustrated in Figure 2b, which depicts the complete light path including laser excitation, scattering collection, spectral dispersion, and signal conversion (Fang et al., 2024). As shown in Figures 2b-ii, conventional laboratory-based Raman systems integrate the full optical setup and are primarily utilized for *ex vivo* and *in vitro* analyses of excised tissues or biopsy samples. In contrast, Figures 2b-iii presents a compact fiber-optic Raman probe, which enables minimally invasive, *in vivo*, and intraoperative real-time detection for clinical applications such as surgical navigation (Auner et al., 2018).

The core advantage of RS lies in its ability to generate a unique biochemical fingerprint of biological tissues: each biomolecule and chemical bond has a specific Raman shift peak. As a non-invasive and label-free analytical tool, RS has been widely used in the field of tumor diagnosis due to its advantages of non-destructiveness, rapidity and high chemical specificity (Kong et al., 2015; Das et al., 2017). It can realize the differential diagnosis of tumor tissue and normal tissue by identifying the changes of biomolecular composition in tumor tissue (such as increased nucleic acid content, decreased lipid content, and abnormal protein structure) (Surmacki et al., 2013). In breast cancer research, Raman imaging technology has reached a clinically relevant level, capable of accurately distinguishing cancerous tissue from normal tissue at safe margins. As shown in Figure 2c, by imaging the intensity of characteristic peaks such as 1,518 cm^−1^ (carotenoids) and 2,854 cm^−1^ (oleic acid), the spatial distribution differences between non-cancerous tissue (carotenoids-rich) and cancerous tissue (with increased protein contribution) can be visually observed. This biochemical fingerprint change is the core basis for determining pathological states (Surmacki et al., 2013). Haka et al. further demonstrated through *in vivo* experiments that the Raman system can perform tissue characterization in just 1 s and can even detect micro cancerous lesions invisible to the naked eye, thereby preventing patients from undergoing secondary surgery due to incomplete margins (Haka et al., 2006).

In liver cancer diagnosis, combined with deep learning algorithms, RS has achieved a transition from laboratory analysis to automated clinical diagnosis. By constructing an intelligent diagnostic workflow, and using convolutional neural networks (CNNs) to automatically extract features from the collected spectra, not only can rapid differentiation between liver cancer tissue and normal tissue be achieved, but tumor subtype identification, differentiation grading, and staging evaluation can also be further performed (Huang et al., 2023). Furthermore, as demonstrated in Figure 2d, the integration of spectral clustering algorithms and imaging techniques allows for the visualization of molecular composition at sub-micrometer resolution (Figures 2d-ii), facilitating precise tumor boundary recognition through the comparison of spectral fingerprints between carcinoma and paracarcinoma regions (Figures 2d-iii) (Huang et al., 2023). Additionally, for thyroid cancer, multimodal fusion techniques (such as combining Raman with FTIR) utilize the complementary molecular information provided by the two spectroscopies, significantly improving the diagnostic accuracy of malignant tumor metastasis (Song et al., 2024).

Although RS performs well in identifying tumors in solid organs, its application in the complex lymphatic system holds greater clinical value. Since LNs are affected by various pathological conditions such as infection, hyperplasia, and tumor metastasis, accurately identifying secondary metastatic lesions is crucial. This review systematically explores the evolution of Raman spectroscopy as a transformative tool for SLN management. Driven by its exceptional chemical specificity, Raman scattering has already established its prowess in high-fidelity biopsy and cell sorting, making its integration into lymphatic diagnostics a natural and necessary evolution toward precision oncology. This section first covers the landscape of label-free detection and the adaptation of conventional clinical tracers, outlining how endogenous molecular signatures can be leveraged to enhance clinical accessibility without the immediate requirement for exogenous contrast agents. The discussion then details the advancements in SERS, where noble metal nanostructures facilitate a dramatic signal leap, enabling the detection of trace biomarkers that are otherwise invisible. To address the inherent constraints of traditional Raman, specifically regarding tissue penetration and acquisition speed, the review discusses the development of advanced derivative and hybrid Raman techniques. These include Surface-Enhanced Resonance Raman Spectroscopy (SERRS) for extreme sensitivity, as well as Surface-Enhanced Spatially Offset Raman Spectroscopy (SESORS) and Surface-Enhanced Transmission Raman Spectroscopy (SETRS) for deep-tissue interrogation. Together, these methodologies represent a sophisticated toolkit for high-performance surgical navigation. Finally, the text summarizes the transition from academic innovation to bedside reality by addressing the challenges of clinical translation, providing a roadmap for transforming these optical advancements into standard-of-care medical practices.

## 2 Conventional Raman spectroscopy in sentinel lymph node biopsy

Conventional RS relies on the inherent inelastic scattering properties of molecules, offering an unperturbed blueprint of the chemical constituents within the lymphatic system. In the context of SLNB, RS has evolved into two complementary clinical strategies based on its unique fingerprinting capability. On one hand, the label-free approach focuses on identifying the subtle pathophysiological changes associated with malignancy, allowing for the direct assessment of intrinsic biomarkers such as nucleic acids, proteins, and lipids to detect occult metastases (Auner et al., 2018; Barkur et al., 2024; Li X. et al., 2023). On the other hand, to surmount the challenges of physical localization and precise margin delineation, novel non-metallic Raman nanotracers with exceptionally large scattering cross-sections have been developed as exogenous labels (Liu et al., 2008; Tian et al., 2020; Gao et al., 2025). These tracers facilitate the real-time identification of SLNs by providing distinct, interference-free spectral signatures.

### 2.1 Direct detection of metastasis-related biomarkers

The core of label-free RS in SLNB is to identify the metastasis status of SLN by analyzing the differences in the content and structure of endogenous biomolecules between metastatic and normal LN tissues. Tumor cell metastasis will lead to significant biochemical changes in SLN: the content of nucleic acids and proteins increases (reflecting the active proliferation of tumor cells), the content of lipids decreases (reflecting the loss of tissue differentiation), and the structure of specific biomolecules (such as keratin, β-carotene) changes (Barkur et al., 2024). These changes can be characterized by the intensity and ratio of specific Raman characteristic peaks (Smith et al., 2003; Som et al., 2016), and the diagnostic model can be constructed by combining multivariate statistical analysis methods [such as principal component analysis (PCA) and linear discriminant analysis (LDA)] to realize the objective diagnosis of SLN (Martina Sattlecker et al., 2010). To explore the clinical utility of this label-free modality, the following subsections examine its application in different types of tumors.

**FIGURE 3:**
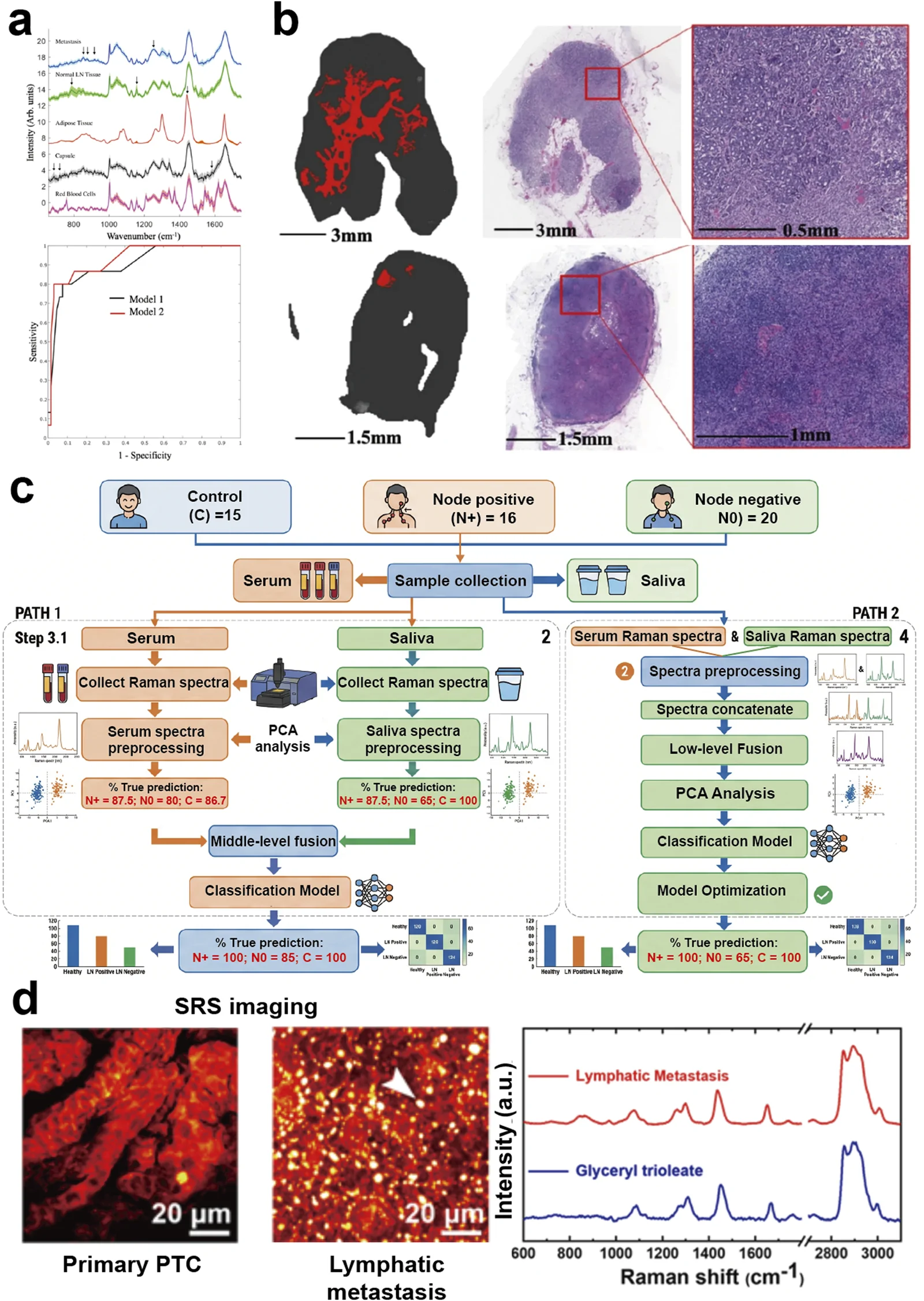
Raman spectral signatures of metastatic biomarkers in breast cancer SLNs across different models and clinical contexts. **(a)** Mean Raman spectra of human metastatic tissue compared with normal lymphoid structures and receiver operating characteristic (ROC) curve for the diagnosis of sentinel lymph node metastasis. Black arrows highlight ten key spectral biomarkers identified for discriminating metastasis, including significant intensity changes in protein and nucleic acid regions (adapted from Barkur et al., 2024, licensed under CC BY 4.0). **(b)** Representative examples of true positive Raman mapping results. The metastatic regions are presented in red in the Raman images. The histology images are presented for comparison (adapted from Barkur et al., 2024, licensed under CC BY 4.0). **(c)** A schematic diagram of the diagnostic methodology for sentinel lymph node metastasis in oral squamous cell carcinoma based on Raman spectroscopy (Note: Original images). **(d)** Stimulated Raman scattering (SRS) images and confocal Raman spectra of lipid droplets in intact human thyroid tissues and lymphatic metastases (adapted with permission from Zeng et al., 2024, Copyright 2024 American Chemical Society) .

Breast cancer is the most extensively studied tumor type for the application of conventional RS in SLNB, with its core mechanism rooted in the significant biochemical alterations in SLNs induced by breast cancer cell metastasis (Barkur et al., 2024; Som et al., 2016). Building upon the specific biochemical variations, recent studies have successfully extracted and validated these spectral signatures across various biological models. As demonstrated in Figure 3a, Barkur et al. identified 10 key spectral features that provide the best discrimination between metastatic and normal human lymphoid tissue structures, directly reflecting the increased protein/nucleic acid content and the depletion of local lipids (Barkur et al., 2024). This biochemical trend is highly consistent in preclinical settings. By establishing a comprehensive spectral library using a mouse mammary tumor model, Thakur et al. verified that normal lymph nodes exhibit predominantly lipid Raman bands, whereas these lipid features (e.g., at 1,748 cm^−1^) almost completely disappear in the metastatic lymph nodes isolated from the tumor-bearing side (Thakur et al., 2010). Furthermore, Som et al. utilized a grid matrix-based tissue mapping protocol to confirm that the high cellular density in completely metastatic lymph nodes leads to both a decreased overall spectral intensity and noticeable shifts in amide and lipid band peaks (Som et al., 2016).

Beyond single-point spectral acquisition, Raman mapping techniques translate these complex biochemical signatures into intuitive spatial visualizations. Smith et al. provided the first description of Raman spectral mapping for axillary lymph nodes by producing false-color spectral images (Smith et al., 2003). As illustrated in Figure 3b, their mapping results distinguish healthy and cancerous tissues based on structural biochemical distribution: normal lymph nodes are characterized by high lipid intensities in central regions (Figures 3bi–iii), while metastatic areas are clearly delineated by the localized accumulation of carotenoids (Figures 3b iv–vi) (Smith et al., 2003). Advancing this spatial evaluation, further research employed confocal Raman spectral mapping to identify local variations of chemical constituents, successfully visualizing even occult micrometastases within the nodal architecture (Smith et al., 2004).

Given the inherent complexity of biological matrices, translating these spectral and spatial features into reliable clinical diagnoses relies heavily on multivariate statistical algorithms. Sattlecker et al. systematically evaluated different Support Vector Machine (SVM) models, revealing that the radial basis function (RBF) SVM achieved a 100% correct classification rate on an independent testing dataset, outperforming traditional linear models (Martina Sattlecker et al., 2010). Recent algorithm innovations have also focused on extracting hidden features from overlapping spectra. For instance, Kim et al. proposed a multimodal biomarker extraction algorithm (PCMA) that couples PCA with multivariate curve resolution-alternating least squares (MCR-ALS), enabling high-efficiency diagnosis when integrated with a portable 785 nm endoscopic fiber-optic probe (Kim et al., 2021). Moving beyond immediate intraoperative diagnosis, label-free RS has demonstrated unprecedented value in prognostic evaluation. Depciuch et al. investigated the biochemical similarity between a patient’s primary breast cancer tissue and their corresponding metastatic lymph nodes to predict postoperative outcomes (Depciuch et al., 2020).

Beyond these well-documented applications in breast cancer, the clinical potential of conventional Raman spectroscopy is also being explored in the sentinel lymph node assessment of other solid tumors. The diagnostic capability of Raman spectroscopy is particularly valuable in the head and neck region, where lymphadenopathy can originate from various complex etiologies, including reactive hyperplasia, primary lymphomas, and secondary metastases from squamous cell carcinomas. Lloyd et al. conducted a comprehensive study analyzing 103 lymph nodes from patients undergoing neck dissection (Lloyd et al., 2013). Utilizing 830 nm Raman spectroscopy combined with multivariate classification models, they successfully distinguished between benign reactive nodes, primary malignancies (Hodgkin’s and non-Hodgkin’s lymphomas), and secondary metastatic carcinomas (Lloyd et al., 2013). The distinct separation of these diverse pathologies in the LDA scatter plots (Figure 3c) underscores the broad applicability of Raman spectroscopy in resolving complex nodal diagnostics (Lloyd et al., 2013). Building on this discriminatory power, Rau et al. mapped the biochemical topography of lymph nodes affected by different conditions, successfully generating false-color Raman images that correspond closely with standard H&E histology. Their work demonstrated that the technique could differentiate a low-grade primary tumor (follicular lymphoma) from both benign hyperplasia and secondary metastases (Rau, 2019).

In the management of thyroid cancer, assessing cervical lymph node metastasis traditionally relies on intraoperative frozen sections or preoperative imaging. Recent advancements in stimulated Raman scattering (SRS) microscopy have enabled rapid, high-resolution evaluation of lymphatic metastasis in papillary thyroid carcinoma (PTC) (Junjie et al., 2024). By integrating multicolor SRS imaging with confocal Raman spectroscopy, Zeng et al. quantitatively analyzed intact human thyroid tissues *in situ* without processing or labeling (Junjie et al., 2024). As shown in Figure 3d, their study identified an aberrant accumulation of triglycerides (TGs) specifically within the lymphatic metastases, a feature absent in normal thyroid tissue, primary PTC, or benign lymph nodes. This specific lipid metabolic alteration serves as a robust signature for PTC nodal metastasis.

Similarly, in gastrointestinal oncology, Raman spectroscopy has been employed to evaluate esophageal cancer lymph nodes. Isabelle et al. utilized Raman mapping to probe the biochemical differences between benign and cancerous esophageal nodes (Isabelle et al., 2008). Their analysis revealed that metastatic nodes exhibit higher nucleic acid content and lower lipid and carbohydrate levels compared to benign nodes, reflecting increased cellular proliferation and loss of differentiation (Isabelle et al., 2008). Using principal component analysis fed into a linear discriminant analysis (PCA-LDA) model, they achieved a high diagnostic accuracy, visually supported by Raman intensity maps and LDA histograms (Isabelle et al., 2008). To translate these benchtop findings into surgical practice, current clinical trials, such as the DOLOMITE study, are actively investigating the *in vivo* application of Raman needle probes (RNP) for real-time identification of malignant deposits in resected esophageal lymph nodes (Ireland et al., 2022). These parallel explorations confirm that the biochemical fingerprinting provided by label-free Raman spectroscopy is a versatile and highly accurate tool for intraoperative nodal staging across multiple oncological disciplines.

### 2.2 Sentinel lymph node biopsy based on novel nanotracers

Although traditional lymphatic mapping agents have been implemented clinically, they pose distinct operational constraints; small-molecule blue dyes suffer from rapid diffusion that leads to non-specific staining of second-tier nodes, near-infrared fluorescent agents face photobleaching challenges, and radioactive isotopes introduce systemic radiation risks and logistical burdens. To circumvent these clinical limitations, the development of novel Raman-active nanotracers is essential to overcome the rapid diffusion and high background interference associated with traditional dyes. These advanced materials are engineered for high biosafety and giant Raman scattering cross-sections, enabling high-contrast identification of SLNs.

**FIGURE 4:**
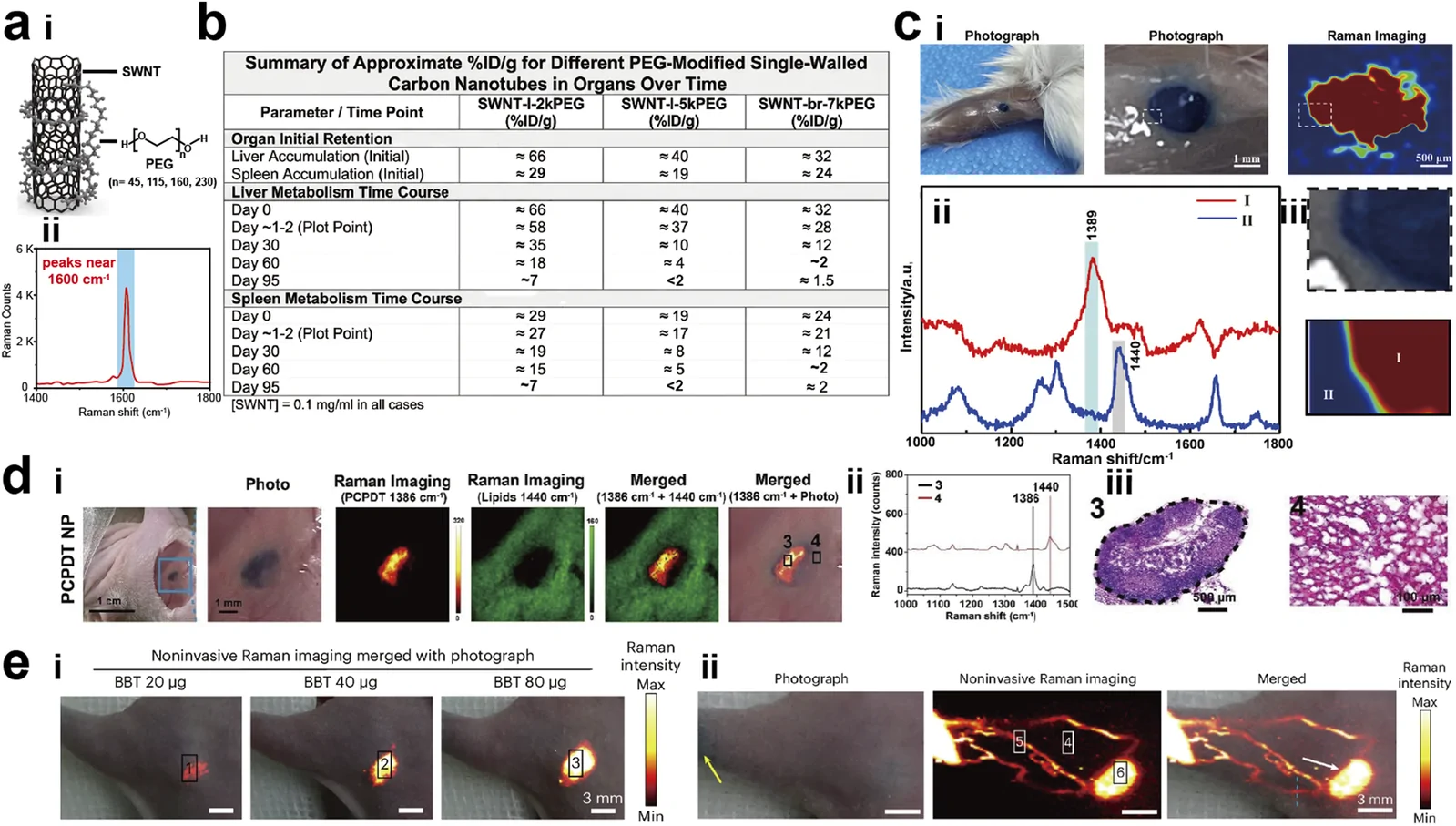
Representative *in vivo* SLN imaging and characterization of labeled Raman probes for SLNB. **(a)** Characterization of PEGylated SWNTs: (i) schematic of noncovalent PEG functionalization for improved biocompatibility and circulation stability; (ii) Raman spectrum showing a specific G-band peak at 1590 cm^−1^ as the labeled signal for *in vivo* detection (Note: Original images). Raman-tracked biokinetics of PEGylated SWNTs in mice: 1 day biodistribution across major organs; 3 months clearance profiles in the liver and spleen. The results highlight that branched PEG functionalization (SWNT-br-7kPEG) significantly minimizes long-term organ retention compared to linear counterparts, facilitating safer lymphatic mapping (Note: Original images). **(c)** In situ SLN imaging of CDT-TT NPs (polymer-based labeled probe): Photographs, Raman imaging, and spectral comparison of mouse popliteal SLNs, showing specific 1389 cm^−1^ Raman signal (red, region I) in SLNs and no interference in adjacent adipose tissue (blue, region II), achieving high-resolution edge visualization (adapted with permission from Bao et al., 2024, Copyright 2024 American Chemical Society). **(d)** Intraoperative background-free Raman imaging of LNs using PCPDT nanoprobes (i), along with corresponding spectral (ii) and histopathological validations (iii) (adapted with permission from Cui et al., 2022, Copyright 2022 American Chemical Society). (e) Noninvasive transdermal SLN and lymphatic imaging of BBT NPs: (i) dose-dependent SLN imaging; (ii) visualization of the lymphatic drainage pathway from injection site to SLNs, demonstrating the clinical translation potential of noninvasive labeled Raman imaging (adapted from Gao et al., 2025, licensed under CC BY 4.0).

Carbon-based nanomaterials, particularly single-walled carbon nanotubes (SWNTs), were among the first to be exploited as non-metallic Raman labels for lymphatic mapping. Liu et al. exploited the intrinsic, high-intensity G-band (1590 cm^−1^) of single-walled carbon nanotubes (SWNTs) for long-term lymphatic mapping (Figure 4a). A critical advancement in this work was the non-covalent functionalization with branched polyethylene-glycol (PEG) chains, which extended the blood circulation half-life to approximately 24 h, as illustrated in Figure 4b. This prolonged circulation allows for the gradual and thorough accumulation of tracers in the lymphatic system. *Ex vivo* Raman mapping of organ slices provides high-resolution spatial distribution data, revealing that while SWNTs primarily accumulate in the reticuloendothelial system (RES), they are gradually cleared through biliary and renal pathways over a 3-month period without inducing long-term toxicity (Liu et al., 2008). Furthermore, the clinical safety profile of carbonaceous tracers has been validated through the study of carbon nanoparticles suspension injection (CNSI). Xie et al. utilized RS to demonstrate that clinical-grade CNSI, despite its accumulation in the liver and spleen, causes only transient, reversible oxidative stress at the 7-day mark, confirming its robust biosafety for preoperative SLN mapping (Ping et al., 2017).

Beyond carbon materials, molecularly engineered donor-acceptor (D-A) conjugated polymers have emerged as potent probes for high-contrast SLN imaging. Bao et al. developed a CDT-TT conjugated polymer probe composed of cyclopenta[2,1-b:3,4-b']dithiophene (CDT) and thieno[3,4-b]thiophene (TT) units, which generates a resonance Raman (RR) effect by matching its absorption band with the 785 nm excitation wavelength. This strategic alignment amplifies the characteristic peak at 1,389 cm^−1^, which is strategically positioned away from the strong 1,440 cm^−1^ lipid vibrational peak of fat. As shown in Figure 4c, this technology allows for the precise demarcation of SLN boundaries within lipid-rich environments, achieving a signal-to-background ratio significantly superior to traditional fluorescence-based mapping (Bao et al., 2024). To effectively overcome the biological background interference inherent to adipose-rich lymphatic environments, Cui et al. developed polycyclopenta[2,1-b;3,4-b’]dithiophene (PCPDT) nanoparticles *via* a molecular planarization strategy (Cui et al., 2022). This structural engineering induced a significant blueshift of the intrinsic Raman peak to 1,386 cm^−1^, successfully bypassing the strong endogenous lipid signal at 1,440 cm^−1^. As illustrated in Figure 4d, this background-free design enabled high-contrast intraoperative Raman imaging of LNs (Figures 4d-i), with its specific accumulation and precise optical localization definitively corroborated by corresponding spectral analysis (Figures 4d-ii) and histopathological validation (Figures 4d-iii) (Cui et al., 2022).

A paradigm shift in labeled RS was further achieved by the introduction of stacking-induced charge-transfer-enhanced Raman scattering (SICTERS). Gao et al. designed substrate-free nanoprobes composed of self-stacked small molecules with bis-thienyl-substituted benzobisthiadiazole nanoparticles (BBT NPs). Through an ordered spatial arrangement that facilitates three-dimensional charge transfer, these nanoprobes achieved a Raman scattering cross-section 1,350 times higher than that of conventional gold SERS nanoprobes of similar size. As illustrated in Figure 4e, this breakthrough allowed for the non-invasive Raman imaging of lymphatic vasculatures and the intraoperative detection of microtumors as small as 0.25 mm (Gao et al., 2025). Collectively, these advancements in labeled RS offer a powerful and biosafe alternative to traditional labeling techniques, providing clinicians with high-contrast molecular feedback for precise SLN assessment.

### 2.3 Advantages and limitations of Raman spectroscopy in sentinel lymph node biopsy

The primary clinical advantage of conventional RS in SLNB lies in its non-destructive, label-free capacity to provide intrinsic “biochemical fingerprints” of tissues (Auner et al., 2018; Rau, 2019). This enables the rapid, intraoperative assessment of fresh LN specimens without compromising their structural integrity, ensuring seamless compatibility with subsequent gold-standard histopathological validation (Barkur et al., 2024; Horsnell et al., 2012; Hubbard et al., 2020). Additionally, the advent of novel non-metallic nanotracers has provided robust, photostable alternatives for long-term lymphatic mapping with exceptional biosafety (Liu et al., 2008; Gao et al., 2025).

Despite these merits, the widespread clinical adoption of conventional RS is fundamentally hindered by the inherently weak inelastic scattering cross-section of biological molecules (Das et al., 2017). This often leads to low signal-to-noise ratios, an issue frequently exacerbated by strong tissue autofluorescence that can easily obscure the subtle spectral peaks of occult micrometastases (Auner et al., 2018; Das et al., 2017; Horsnell et al., 2012). To overcome these fundamental limitations in sensitivity and background interference, researchers have increasingly turned to plasmonic enhancement strategies, leading to the development of SERS.

## 3 SERS in sentinel lymph node biopsy

**FIGURE 5:**
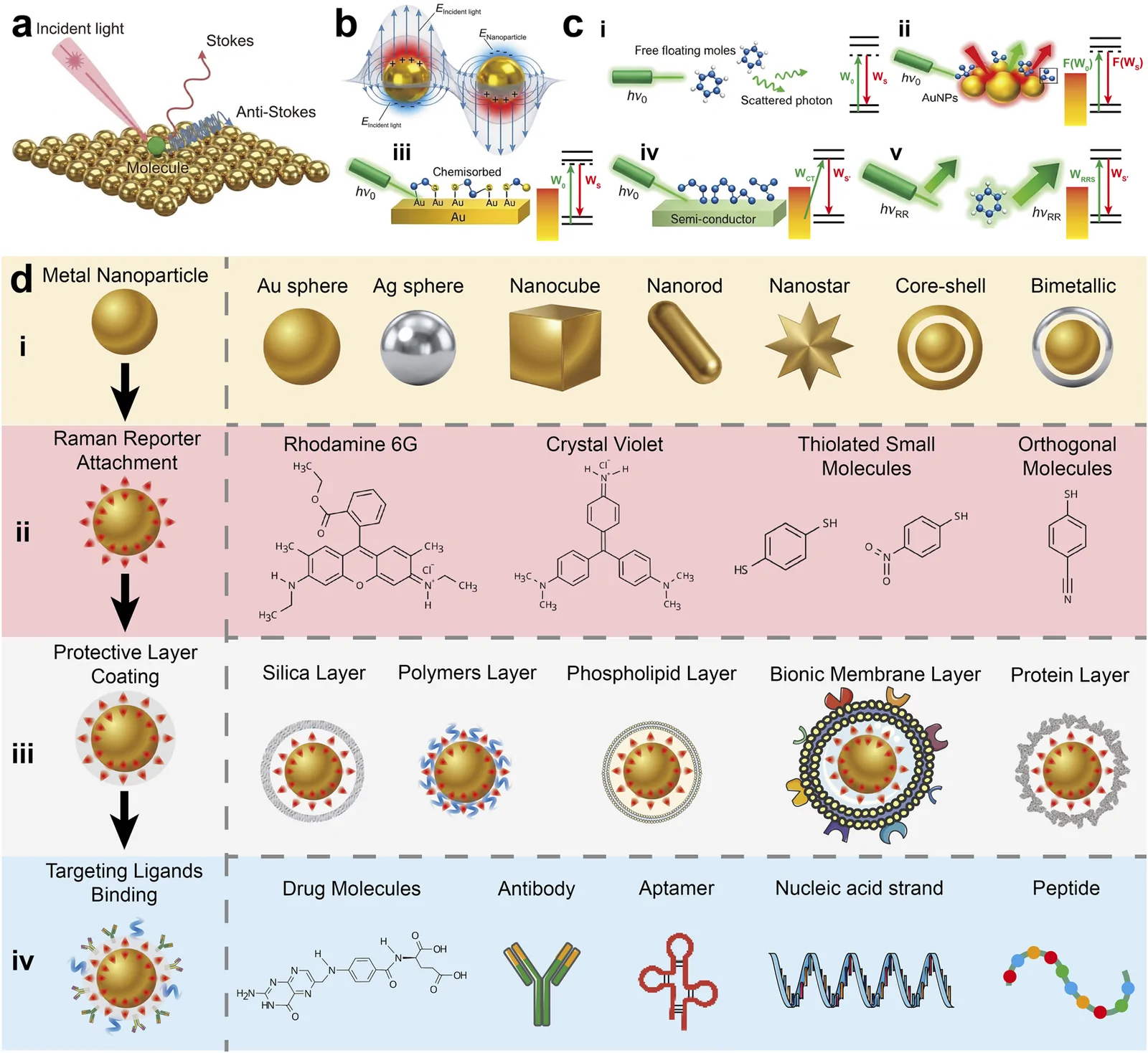
Physical and chemical principles of signal enhancement in SERS and preparation process of SERS nanotags. **(a)** SERS signals generated by molecules adsorbed on gold nanostructures, involving both Stokes and anti-Stokes Raman scattering processes. **(b)** LSPR plays a crucial role in achieving SERS effects. **(c)** Schematic diagram (i) depicts conventional normal Raman scattering (RS). The distinct enhancement mechanisms underlying SERS are illustrated as follows: the electromagnetic enhancement mechanism (EM) in (ii), the chemical enhancement mechanism (CM) in (iii), the charge-transfer mechanism (CT) in (iv), and the resonance Raman scattering mechanism (RRS) in (v). **(d)** Typical preparation process of SERS nanotags: (i) noble metal nanoparticle core, (ii) Raman reporter adsorption, (iii) protective shell coating, and (iv) targeting ligand functionalization.

SERS is a high-performance derivative of conventional RS that fundamentally overcomes the inherent limitation of weak Raman scattering in biological detection (Constantinou et al., 2022). As illustrated in Figure 5a, the process begins when a target molecule adsorbs onto a noble metal surface; upon laser excitation, the molecule emits significantly amplified Stokes and anti-Stokes Raman scattering signals. As comprehensively reviewed by Constantinou et al. (2022), the SERS effect is primarily driven by two synergistic mechanisms: electromagnetic enhancement (EM) and chemical enhancement (CM). The EM effect arises from the localized surface plasmon resonance (LSPR) of noble metal nanostructures. As shown in Figure 5b, the incident light’s electric field (E _Incident light_) triggers a collective oscillation of conduction electrons within the nanoparticle, generating a powerful secondary electric field (E _Nanoparticle_). This resonance creates “hotspots”, regions of intense local electric fields at nanogaps or sharp curvatures, capable of amplifying Raman signals by up to 10^10^ times, which is EM effect (Figures 5c-ii). Complementary to EM, the CM effect is a multi-factor contribution, primarily including charge transfer (CT) and resonance Raman (RRS) mechanisms (Figures 5c-iii) (Constantinou et al., 2022; Qian et al., 2008). Specifically, the CT mechanism involves the exchange of electrons between the metal’s Fermi level and the molecular orbitals facilitated by incident photons (Figures 5c-iv), while the RRS effect occurs when the excitation frequency matches the electronic transition of the molecule (Figures 5c-v).

In clinical practice, SERS encompasses two core strategies: label-free detection for direct acquisition of endogenous biomolecular fingerprints, and labeled detection utilizing targeted nanoprobes for specific molecular recognition. The pioneering development of SERS nanoparticle tags achieved the first *in vivo* tumor targeting and spectroscopic detection (Qian et al., 2008), establishing a foundational paradigm for the use of functionalized nanoprobes in cancer diagnostics. Given their proven success in systemic tumor imaging, these SERS-active tags have been naturally extended to the field of lymphatic mapping. High-performance SERS nanoprobes (nanotags) are essential for precise SLN localization. As shown in Figure 5d, a typical nanotag consists of four functional layers: a plasmonic metal core for electromagnetic signal amplification, and a Raman reporter layer providing distinct vibrational fingerprints. To maintain signal integrity in complex lymphatic environments, an encapsulation shell is employed to prevent reporter leakage and particle aggregation. Finally, an outer functionalization layer dictates the biological identity of the probe; by incorporating specific ligands, it ensures selective accumulation within lymphatic targets. This integrated architecture provides the necessary optical brightness for deep-tissue tracking while enabling the highly accurate discrimination of SLNs from surrounding healthy tissues and non-specific biological backgrounds.

The metallic core serves as the fundamental engine of a SERS nanotag, primarily functioning to amplify the Raman scattering signals of the adsorbed reporter molecules through the localized LSPR effect (Figures 5d-i). Historically, noble metals such as gold and silver have been the predominant choices for core materials due to their exceptional dielectric properties (Fabris, 2015; Tong, 2018). While gold offers superior chemical stability and biocompatibility, silver typically yields higher enhancement factors but suffers from susceptibility to oxidation. To leverage the strengths of both, bimetallic nanostructures (e.g., Au@Ag or Au-Ag alloys) have emerged as an effective strategy to achieve both robust signal intensity and long-term stability (Fan et al., 2013). Furthermore, recent studies have demonstrated that non-metallic two-dimensional materials also possess noteworthy enhancement capabilities, expanding the material library beyond traditional noble metals (Son et al., 2021). Beyond material composition, the geometric morphology of the core, ranging from nanospheres (Wang et al., 2005) and nanorods (Zhang et al., 2013) to branched nanostars (Indrasekara et al., 2014) or nanoflower (Zhang et al., 2019; Deng et al., 2022), critically dictates the LSPR wavelength. By precisely tuning these shapes, the plasmonic resonance can be tailored to match specific excitation lasers, thereby maximizing the generation of electromagnetic “hotspots” and optimizing the overall sensitivity for SLN tracing.

The Raman reporter is the strategic component of a SERS nanotag, responsible for providing high-specificity spectral signatures that enable the precise differentiation of the SLN from surrounding healthy tissues. To ensure detectable signals in complex biological environments, the primary requirement for a reporter is a large Raman scattering cross-section. These molecules are generally categorized into three classes, as shown in Figures 5d-ii: fluorescent dyes (Kneipp et al., 2009), thiol-containing organic molecules (Gu et al., 2020), and bio-orthogonal molecules (Lin et al., 2023). While these categories share the commonality of producing intense vibrational signals, each possesses distinct trade-offs. Fluorescent molecules offer exceptionally high signal intensity but are often plagued by strong fluorescence backgrounds that can interfere with Raman peaks. Thiol-containing small molecules form stable chemical bonds with noble metal cores, facilitating robust probe construction; however, their spectral fingerprints often overlap with the endogenous signals of biological tissues, limiting their specificity. In contrast, bio-orthogonal molecules provide an “optical window” by vibrating in the biologically silent region (1,800–2,800 cm^−1^), effectively eliminating background interference. Nevertheless, many commercial bio-orthogonal reporters lack thiol groups for strong metal adsorption and typically exhibit lower intrinsic scattering intensities compared to traditional dyes. Consequently, the selection of an ideal reporter must be meticulously balanced with the geometric control of the plasmonic core to achieve optimized signal-to-noise ratios and meet the specific demands of intraoperative SLN detection.

While metallic cores and reporters provide the necessary signal, they cannot be used directly in complex biochemical environments due to potential reporter dissociation and nanoparticle aggregation. Surface encapsulation is essential to protect the SERS nanotags from physiological interference, enhance biocompatibility, and reduce toxicity (Figures 5d-iii) (Liu et al., 2022). Various materials, including silica (Deng et al., 2022; Deng et al., 2024b), polymers (Rao and Radhakrishnan, 2015), biomolecules, and metal-organic frameworks (MOFs) (Sun et al., 2018), have been developed as protective shells. Silica is a classic choice, where thickness can be precisely tuned using precursors like sodium silicate for ultrathin layers or TEOS for robust, thick coatings (Deng et al., 2022; Deng et al., 2024b). Polymers, most notably SH-PEG, are widely utilized to prevent aggregation while providing functional end-groups for subsequent ligand attachment (Feng et al., 2017; Wu et al., 2023). Recently, polydopamine (PDA) has emerged as a versatile shell that allows for one-pot synthesis and subsequent Michael addition with targeting moieties (Lin et al., 2023; Li et al., 2022). Biomolecules such as bovine serum albumin (BSA) and phospholipids are also employed to mimic biological interfaces, with phospholipids specifically offering self-assembly capabilities and the potential for core-satellite structural engineering (Höller et al., 2020; Zheng et al., 2019). Furthermore, advanced shells like MOFs (e.g., ZIF-8) and coordination polymers (e.g., Prussian Blue) have gained attention (Sun et al., 2018; Yin et al., 2017). These materials not only act as molecular filters to enhance sensing specificity but can also provide intrinsic, background-free signals in the Raman-silent region due to abundant cyano (C≡N) groups (Yin et al., 2017). Ultimately, the choice of encapsulation material dictates the probe’s robustness and its ability to maintain high signal-to-noise ratios during the dynamic process of lymphatic transport and SLN mapping.

To achieve precise SLN localization, the outermost surface of SERS nanotags must be functionalized with biorecognition molecules, such as antibodies, peptides, oligonucleotides, or aptamers (Wang et al., 2024), to ensure high specificity and affinity for target biomolecules (Figures 5d-iv). These ligands can be immobilized through various strategies depending on the probe’s architecture. For direct core modification, sulfhydryl-containing ligands can be attached *via* robust Au-S or Ag-S bonds, though this may involve competition with existing protective molecules (Liu et al., 2022). Alternatively, to maintain the integrity of the encapsulation shell, ligands are frequently conjugated to the outer layer *via* chemical cross-linking. A common approach involves one-ethyl-3-(3-(dimethylamino)propyl) carbodiimide (EDC) and N-hydroxysuccinimides (NHS) (EDC/NHS) chemistry to form stable amide bonds between carboxylic acid and primary amine groups, effectively immobilizing proteins and peptides (Pal et al., 2017; Wei et al., 2021). Other effective strategies include leveraging the high-affinity biotin-streptavidin system, utilizing electrostatic interactions between oppositely charged species (e.g., poly-L-lysine), or forming disulfide (S-S) bonds with cysteine-terminated peptides on BSA-coated surfaces (Zheng et al., 2019; Yin et al., 2017). This functionalization not only dictates the biological identity of the nanotags but also facilitates their selective accumulation within the SLN, minimizing non-specific background signals during intraoperative tracing.

### 3.1 Label-free application form

**FIGURE 6:**
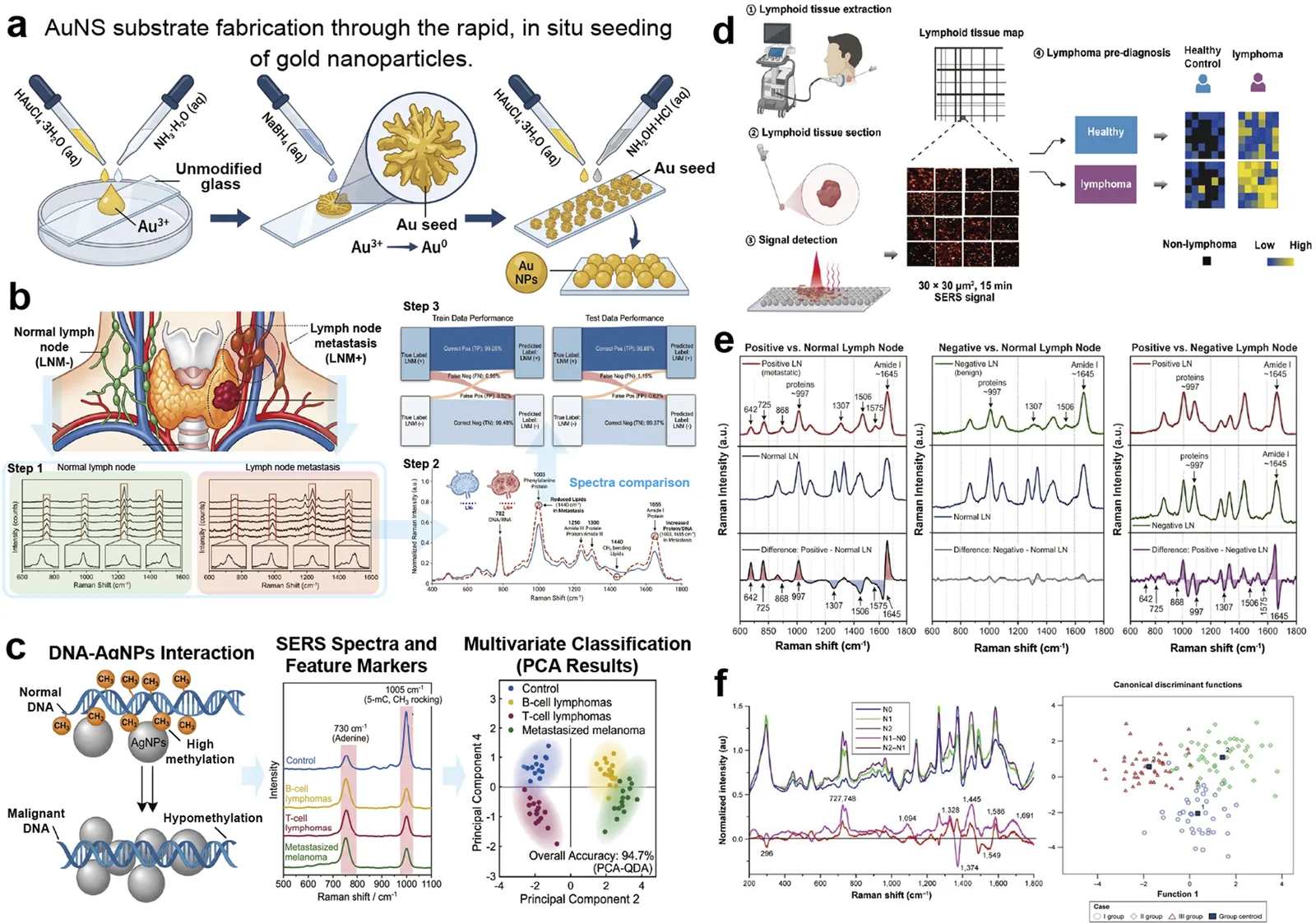
Label-free SERS platforms and diagnostic frameworks for SLN assessment. **(a)** Schematic traditional procedure for the fabrication of AuNS substrates via rapid, in situ seeding of gold nanoparticles (Note: Original image). **(b)** The framework for AI-assisted diagnosis of papillary thyroid carcinoma consists of three steps: Step 1, acquisition of Raman spectra of normal lymph node samples and lymph node metastasis samples; Step 2, comparison of mean SERS of normal tissue and lymph node metastasis samples obtained by fine-needle aspiration and identification of potential differentially expressed molecules; and Step 3, demonstration of robust classification performance through confusion matrices of the training and test sets (Note: Original image). **(c)** Identification of B-cell/T-cell lymphomas and metastasized melanoma based on SERS-detected DNA methylation patterns (Note: Original image). **(d)** Schematic of a silicon-based SERS imaging platform coupled with deep learning for the screening of clinical lymphatic sections (reproduced from Cao et al. 2025, licensed under CC BY-NC-ND 4.0). **(e)** Average and difference SERS spectra of urinary modified nucleosides from LN with (LN+) or without metastasis (LN-) and healthy controls (C), highlighting the potential for indirect metastasis monitoring (Note: Original image). **(f)** Representative SERS spectra across different nodal stages (N0-N2), difference spectra, and canonical discriminant analysis, showcasing the capability of SERS for precise pathological staging (adapted from Xue et al. 2018, licensed under CC BY-NC).

Label-free SERS enables direct capture of intrinsic molecular vibrational fingerprints from biological samples without exogenous Raman reporters or targeted probes, and has become a pivotal technique for preoperative evaluation and intraoperative screening of SLN metastasis due to its non-invasive, rapid and label-free merits. In a representative study focused on papillary thyroid carcinoma (Figures 6a,b) (Hou et al., 2025), researchers developed a high-performance solid-phase SERS platform to streamline the assessment of SLN status. The workflow began with the optimization of gold nanostructure (AuNS) substrates through a seed-mediated growth strategy, ensuring high reproducibility and electromagnetic enhancement for label-free detection (Figure 6a). By integrating SERS with advanced machine learning, the team successfully analyzed clinical samples obtained *via* fine-needle aspiration (Figure 6b). As shown in Figures 6b–6ii, the resulting SERS spectra revealed distinct biochemical signatures, where metastasized LNs exhibited characteristic shifts in protein and nucleic acid vibrations compared to normal lymphatic tissues. To handle the complexity of these endogenous fingerprints, a PCA-LDA classification model was employed. The diagnostic robustness was validated by confusion matrices (Figures 6b-iii), demonstrating exceptional accuracy in both training and independent test sets. Beyond direct tissue scanning, SERS technology has been successfully applied to the molecular profiling of extracted genomic DNA for SLN evaluation (Figure 6c) (S et al., 2021). In this approach, DNA is isolated from LN biopsies to identify specific epigenetic markers associated with malignancy. As shown in Figure 6c (S et al., 2021), the SERS spectra exhibit distinctive vibrational fingerprints at 730 cm^−1^ (adenine ring breathing) and 1005 cm^−1^ (phenylalanine or specific DNA backbone modes), which serve as critical indicators for distinguishing metastatic involvement. By capturing these spectral variations, the platform achieved high-precision classification of various clinical conditions, including B-cell lymphomas, T-cell lymphomas, and metastasized melanoma. This DNA-based SERS analysis offers a robust molecular diagnostic layer, providing high specificity for identifying metastatic origins at the nucleic acid level. While SLNB traditionally relies on histopathological staining, emerging SERS imaging techniques offer a label-free alternative for identifying malignancy within lymphoid structures. For instance, another significant work introduced a silicon-based SERS chip for the direct imaging analysis of lymphoid tissue sections (Figure 6d) (Cao et al., 2025). Unlike the DNA-based profiling in Figure 6c, this approach performs label-free scanning on intact tissue slices obtained *via* biopsies. This methodology aligns closely with clinical immunohistochemistry (IHC) but offers a more rapid, chemical-specific alternative that requires no complex staining procedures. As shown in Figure 6d, the analytical workflow entails three primary stages: (i) extraction and sectioning of lymphoid tissues; (ii) high-throughput SERS signal acquisition using a large-area plasmonic chip; and (iii) automated diagnostic mapping. To manage the vast amount of spectral data generated during imaging, a deep learning framework was implemented to differentiate between healthy controls and various lymphoma subtypes. The results demonstrated that the SERS imaging maps could accurately visualize the distribution of malignant areas within the tissue. Although this study primarily focused on lymphoma diagnosis, the underlying methodology for visualizing cancerous infiltration within the LN architecture is highly translatable to the rapid assessment of metastatic involvement in SLNB.

Beyond tissue-level examinations, analyzing biochemical information in biofluids provides a non-invasive alternative for evaluating LN status. In studies focused on determining metastatic involvement, SERS was utilized to capture systemic changes associated with LN metastasis. Specifically, by monitoring urinary modified nucleosides, researchers successfully established a correlation between spectral profiles and the presence of LN metastasis (Figure 6e) (Lin et al., 2024). Complementary to this, serum-based SERS analysis demonstrated the capability to discriminate among different degrees of nodal involvement, accurately categorizing patients into various pathologic N-stages (e.g., N0, N1, and N2) (Figure 6f) (Lili et al., 2018). In both urinary and serum workflows, as illustrated in Figures 6e,f, the average SERS spectra and their corresponding difference spectra provided clear visualizations of the subtle molecular variations linked to increasing nodal involvement. The urinary-based study leveraged a mid-level data fusion strategy to enhance the preoperative detection of LN metastasis, while the serum-based research developed a robust diagnostic model that effectively identified the progression of lymphatic spread.

In summary, label-free SERS offers a rapid and minimally invasive platform for assessing SLN status by detecting endogenous “fingerprints” in tissues or biofluids. However, the inherent complexity of biological matrices often leads to overlapping spectral signals, which can obscure specific molecular information. To overcome these limitations and achieve higher targeting precision and multiplexed imaging, the development of functionalized SERS nanoprobes has become a vital research frontier, as detailed in the following section on labeled SERS detection.

### 3.2 Labeled application form: precise detection based on targeted SERS nanoprobes

While label-free SERS demonstrates significant potential for the biochemical profiling of lymphatic tissues, its effectiveness in SLN localization and intraoperative detection is often constrained by the low signal-to-noise ratio in deep tissues and the inherent complexity of biological matrices for precise nodal staging. To overcome these limitations, labeled SERS utilizing functionalized nanoprobes has emerged as a superior strategy, providing high-precision targeting, signal amplification, and multiplexed imaging capabilities. In this section, we will systematically discuss the progress of labeled SERS in SLN assessment, focusing on (i) the validation of probe-based SLN localization across various tumor models; and (ii) their integration with handheld Raman systems for real-time intraoperative navigation in large animal models.

Despite the widespread clinical use of traditional tracers for SLN mapping, several inherent limitations persist. Small-molecule dyes, such as methylene blue, suffer from rapid lymphatic drainage beyond the sentinel nodes, often leading to non-specific labeling of second-tier LNs, excessive dissection, and a heightened risk of false negatives. While near-infrared fluorescent agents like indocyanine green (ICG) offer improved visualization, they remain susceptible to photobleaching and the same “over-drainage” issues. Furthermore, radioactive isotopes, the current gold standard, pose radiation risks and necessitate preoperative administration, imposing significant physiological and psychological burdens on both patients and medical staff. In contrast, SERS nanoprobes present a transformative alternative. Due to their tunable particle size, SERS tags exhibit optimized lymphatic transport kinetics that effectively minimize leakage to non-sentinel nodes. Moreover, their unique spectroscopic fingerprints provide high molecular specificity and an ultra-high signal-to-background ratio, ensuring precise localization without the interference of tissue autofluorescence or the hazards of ionizing radiation. These advantages position SERS nanoprobes as a promising candidate for next-generation SLN tracing and intraoperative diagnostics.

**FIGURE 7:**
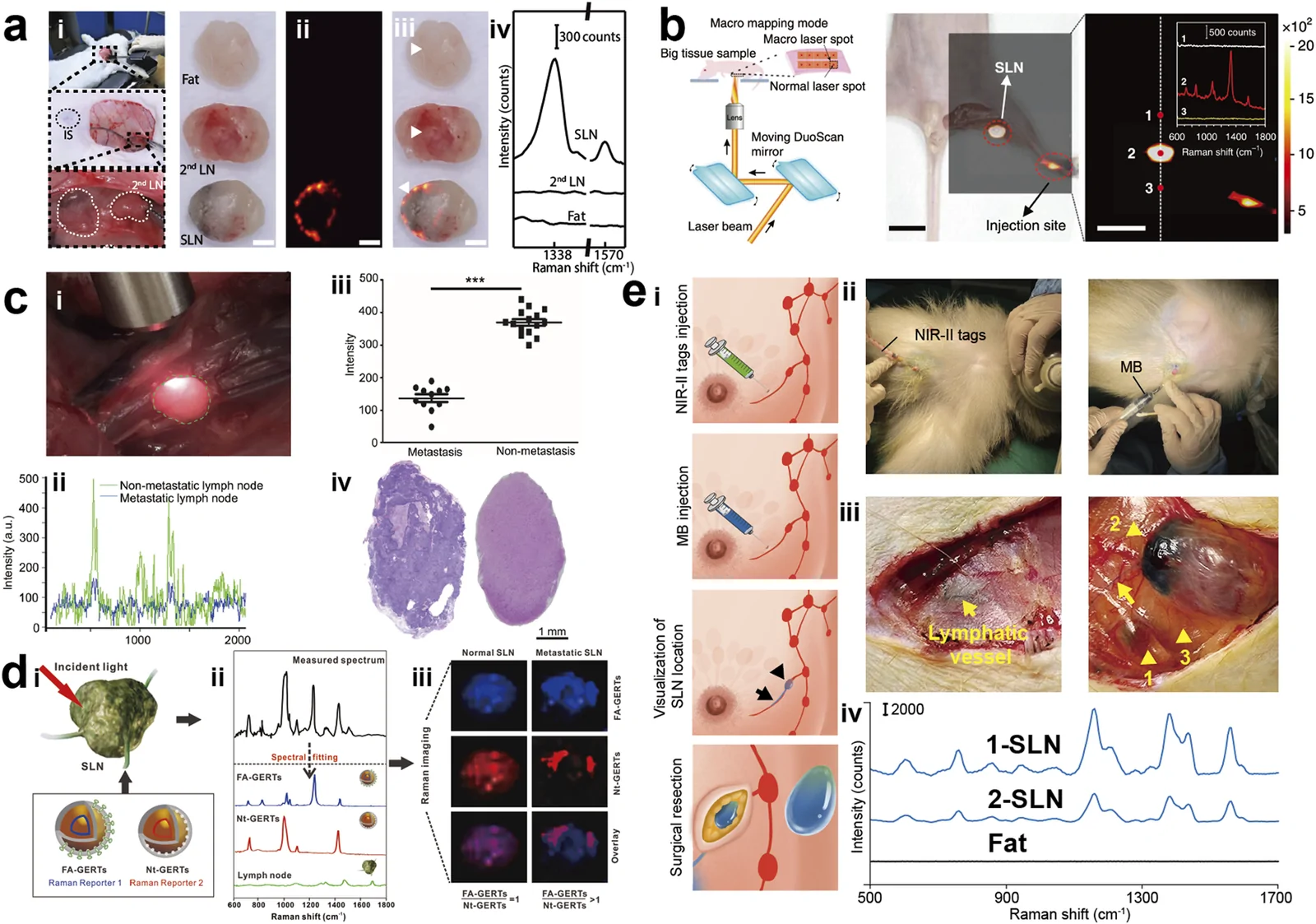
Applications of SERS nanoprobes for *in vivo* SLN localization and metastatic assessment in various tumor models. **(a)** Macroscopic color changes of the SLN, the corresponding SERS mapping and Raman spectra, demonstrating high-resolution discrimination between the SLN, non-SLN (2nd LN) and adjacent adipose tissues (adapted from Deng et al. 2022, licensed under CC BY 3.0). **(b)** Macro-scan mode for wide-area tissue imaging, including in vivo Raman images of hind-limb lymphatic drainage and point-wise spectral comparisons among the SLN (point 2), adjacent tissue (point 1), and background (point 3) (adapted from Zhang et al. 2019, licensed under CC BY 4.0). **(c)** Clinical-simulated SLN identification and metastatic assessment, where SERS spectral variations between metastatic and non-metastatic nodes are validated by gold-standard H&E staining (reproduced from Sun et al. 2019, licensed under CC BY-NC-ND). **(d)** Design of ratiometric Raman nanotags optimized for the intraoperative detection of metastatic SLNs, enhancing diagnostic reliability through signal normalization (adapted from Bao et al., 2021, © 2021 Elsevier Ltd, with permission from Elsevier). **(e)** *In vivo* visualization of breast SLNs in a rhesus macaque model assisted by composite SERS tracers (Note: Original image).

A seminal study published in 2018 established a foundational framework for using SERS nanoparticles as high-performance tracers for SLN mapping (Bao et al., 2018). Following the administration of SERS nanotags *via* footpad injection in a murine model, the targeted SLN could be macroscopically identified by a distinct color change induced by the accumulated nanoparticles (Figures 7a-i). Leveraging the unique and intense spectroscopic signatures of the SERS tags, the researchers achieved high-contrast imaging of the SLN with exceptional spatial resolution, as shown in Figures 7a-ii–vi. The Raman spectra (Figures 7a-iii, vi) clearly demonstrate a sharp distinction between the SERS-labeled lymphatic tissue and the surrounding adipose tissue, which lacks these characteristic vibrational fingerprints. The primary innovation of this work lies in the successful integration of high-brightness SERS nanoparticles with anatomical localization, providing a far superior signal-to-background ratio compared to traditional fluorescence. However, a significant limitation of this pioneering approach was the relatively long acquisition time required for complete SLN mapping. This clinical bottleneck eventually necessitated the development of accelerated scanning strategies, leading to the emergence of high-speed Raman imaging systems. To overcome the clinical bottleneck of slow imaging speeds, Zhang et al. developed ultra-bright, petal-like SERS nanoparticles (GERTs) featuring dense electromagnetic hotspots, which provided the signal intensity necessary for rapid acquisition (Zhang et al., 2019). As illustrated in the schematic (Figure 7b, left), the researchers implemented a high-speed imaging mode utilizing a DuoScan mirror system, which allows the laser focal point to move rapidly across the tissue instead of traditional stage movement. The results demonstrated accurate localization and imaging of the popliteal SLN in murine models (Figure 7b, right). Notably, a wide-area scan of 3.2 × 2.8 cm^2^ was completed in just 52 s (20 × 20 pixels), utilizing an ultra-low laser power of 370 μW and an acquisition time of only 0.7 m per pixel. At the time of publication, this represented the fastest recorded SERS bioimaging speed, significantly advancing the feasibility of real-time intraoperative guidance. Despite this leap in performance, a remaining challenge for clinical translation was the requirement for a darkroom environment during imaging. Furthermore, the intrinsic photoluminescence of certain SERS tags and endogenous tissue background continued to interfere with the signal-to-background ratio, driving the need for new strategies to achieve high-contrast imaging under ambient light conditions. To address the environmental light interference and background noise mentioned previously, Zhu et al. (2022) introduced SERS nanoprobes utilizing bio-orthogonal Raman reporters (as detailed in the Raman reporter subsection of Section 3). By leveraging characteristic signals in the biologically silent region (1,800–2,800 cm^−1^), the researchers achieved high-resolution imaging under ambient light while eliminating interference from the nanoprobe’s intrinsic photoluminescence and tissue autofluorescence. While this work successfully validates the feasibility of SERS nanotags for high-contrast SLN localization and tracing, it primarily focuses on anatomical imaging. The real-time *in vivo* differentiation between metastatic and non-metastatic sentinel LNs remains a challenge, prompting further diagnostic exploration. Building upon localization capabilities, Sun et al. (2019) advanced the field by achieving the *in vivo* diagnostic differentiation between metastatic and non-metastatic SLNs (Figures 7c-i). In this study, SERS nanoprobes were employed to identify nodal status prior to surgical intervention. The SERS signal recorded from metastatic SLNs was remarkably lower than that of non-metastatic nodes (Figures 7c-ii, iii). According to the literature, this phenomenon is attributed to the “mass effect” of tumor proliferation; the dense infiltration of cancer cells disrupts the normal lymphatic architecture and restricts the efficient transport and accumulation of SERS nanoparticles within the nodal cortex. The diagnostic accuracy of the SERS-guided approach was subsequently validated through gold-standard histological analysis (H&E staining, Figures 7c-iv), which showed high correlation with the Raman findings. This study highlights the potential of SERS nanoprobes not only for anatomical tracing but also for providing critical pathological insights during image-guided surgery. As previously discussed, structural alterations within a metastatic SLN significantly impact the accumulation of SERS nanoparticles. However, inflammation-induced nodal swelling can similarly alter nanoprobe behavior, making it difficult to distinguish metastatic from inflammatory nodes using single-signal intensity. To overcome this, Bao et al. (2021) developed a ratiometric strategy employing two types of nanoprobes as shown in Figures 7d-i: a “passive” probe (Nt-GERTs) without targeting ligands and an “active” probe (FA-GERTs) functionalized with folic acid (FA). The diagnostic principle relies on the over-expression of folate receptors on tumor cell surfaces. In a metastatic SLN, FA-GERTs are specifically retained due to high-affinity binding with cancer cells, while Nt-GERTs are gradually metabolized and cleared. Consequently, a signal ratio (Active/Passive) greater than one serves as a reliable indicator of metastasis, whereas a ratio near or below one suggests a non-metastatic or inflammatory state (Figures 7d-ii, iii). This ratiometric approach effectively normalizes for individual variations in lymphatic flow, providing a robust platform for the intraoperative biopsy of SLNs with high specificity.

In summary, these studies collectively demonstrate that SERS nanoprobes can achieve high-contrast anatomical localization and precise metastatic diagnosis within small animal models by optimizing particle morphology, signal-to-background ratios, and ratiometric targeting strategies. To further bridge the gap toward clinical application, recent research has transitioned from benchtop imaging systems to portable handheld Raman devices, enabling real-time intraoperative SLN navigation in large animal models such as rabbits and non-human primates.

In an early demonstration of portable clinical tools, Cha et al. (2017) utilized a handheld Raman device to achieve the accurate *in vivo* localization of SLNs. The SERS-labeled SLN exhibits a distinct macroscopic color change compared to the unlabeled node, a finding further validated by complementary photoacoustic imaging and characteristic Raman spectra. The integration of SERS with photoacoustic modality highlights the technology’s superior multiplexing capabilities and high sensitivity for real-time lymphatic tracing. This pioneering work represents one of the earliest applications of handheld, portable SERS systems for *in vivo* detection, proving that high-performance spectral identification can be moved from the lab bench to the bedside. Moving closer to clinical practice, Spaziani et al. (2023) implemented a SERS-assisted sandwich immunoassay for the detection of human thyroglobulin (Tg) in specimens obtained *via* fine-needle aspiration biopsy (FNAB), a method highly relevant to clinical diagnostic workflows. This platform utilizes a portable optical fiber probe for signal collection, highlighting the potential of flexible and compact detection systems for point-of-care applications. This approach achieves exceptional sensitivity and selectivity, enabling the quantification of trace-level biomarkers within complex human lymphatic samples. While this work validates the feasibility of using handheld fiber-based devices for high-precision assessment of SLN specimens, the detection was performed *ex vivo* post-biopsy. Consequently, there remains a critical need for advanced SERS technologies capable of providing real-time, *in vivo* localization and diagnosis during the surgical procedure itself. To further simulate clinical scenarios, Deng et al. (2022) utilized white New Zealand rabbit models to demonstrate the efficacy of SERS nanotags for SLN tracing. By subcutaneously injecting SERS nanoparticles into the mammary teats of female rabbits, the researchers successfully replicated the entire clinical workflow of human breast cancer SLN mapping. Utilizing a handheld Raman spectrometer, they achieved both non-invasive preoperative percutaneous localization and high-precision intraoperative navigation. A key highlight of this work is the comparative analysis with clinically approved tracers, including methylene blue and indocyanine green. While traditional small-molecule tracers suffer from rapid diffusion to second-echelon LNs, the SERS nanotags provided an extended surgical window of at least 4 h. This temporal stability allows surgeons to accurately differentiate the SLN from downstream nodes, significantly reducing the risk of excessive lymphatic resection and associated side effects.

While SERS nanoprobes demonstrate significant potential for SLN mapping, a major hurdle for clinical translation is that most current studies employ laser power densities far exceeding the Maximum Permissible Exposure (MPE) limits. To address this safety bottleneck, Deng et al. (2024b) developed ultrabright NIR-II SERS nanotags. By utilizing 1064 nm excitation, this work achieved the accurate localization of axillary SLNs in rhesus macaque models for the first time within clinically safe laser dosages. Unlike mice or rabbits, rhesus macaques possess anatomical structures and lymphatic drainage patterns highly analogous to humans, providing a more rigorous validation of the technology’s clinical feasibility. Building upon this photosafe foundation, the researchers further addressed the practical needs of surgeons who prioritize rapid “naked-eye” preliminary localization followed by precise spectral confirmation (Deng et al., 2025). Current clinical practice often relies on a combination of two tracers (e.g., methylene blue, ICG, or radioisotopes) to mitigate the failure of a single agent, yet the inherent limitations of these traditional tracers remain unresolved. In a significant advancement, a novel single-injection composite tracer, as depicted in Figure 7e, was developed by integrating methylene blue with NIR-II SERS nanoprobes. In rhesus macaque models, as depicted in the dual-tracing schematic (Figures 7e-i), the strategy follows a standard clinical injection protocol to ensure its translatability (Figures 7e-ii), the methylene blue (MB) component facilitates immediate visual identification of lymphatic vessels (Figures 7e-iii), while the 1064 nm excited SERS signals provide definitive molecular confirmation of the SLN location (Figures 7e-iv). This synergistic approach effectively combines the intuitive nature of dye-based visualization with the high specificity of RS, offering a highly translatable and precise solution for intraoperative SLN management.

Overall, this section highlights the significant progress of SERS technology in moving from laboratory validation toward clinical feasibility, demonstrating the potential of handheld devices, NIR-II excitation, and composite tracers for safe, real-time, and high-precision sentinel LN management in large animal models.

### 3.3 Advantages and limitations of SERS in sentinel lymph node biopsy

By leveraging electromagnetic and chemical enhancements, SERS fundamentally addresses the sensitivity bottleneck of conventional RS, offering extraordinary detection limits down to the picomolar or picogram-per-milliliter range (Qian et al., 2008; Lou et al., 2026). Coupled with the narrow spectral linewidths characteristic of Raman scattering, SERS minimizes tissue autofluorescence and facilitates the simultaneous multiplexing of several biomarkers (Auner et al., 2018; Cui et al., 2022). Furthermore, targeted SERS nanoprobes exhibit exceptional photostability under prolonged intraoperative laser irradiation, outperforming traditional organic fluorophores and enabling sustained, high-contrast surgical navigation (Cui et al., 2022; Qian et al., 2008).

However, translating SERS from preclinical models to routine clinical use presents distinct challenges. The absolute quantification of SERS signals remains difficult due to poor signal reproducibility, which is highly sensitive to nanoparticle aggregation and the heterogeneous nature of electromagnetic “hotspots” in complex biofluids (Cui et al., 2022; Han et al., 2021). More importantly, despite the amplified signal intensity, conventional SERS imaging is strictly limited by the shallow tissue penetration depth of optical photons (Auner et al., 2018; Cui et al., 2022). To evaluate deep-seated nodes without extensive surgical dissection, advanced Raman geometries capable of macroscopic volumetric sampling and deep-tissue photon extraction are urgently required.

## 4 Derivative Raman technologies for deep-tissue detection

**FIGURE 8:**
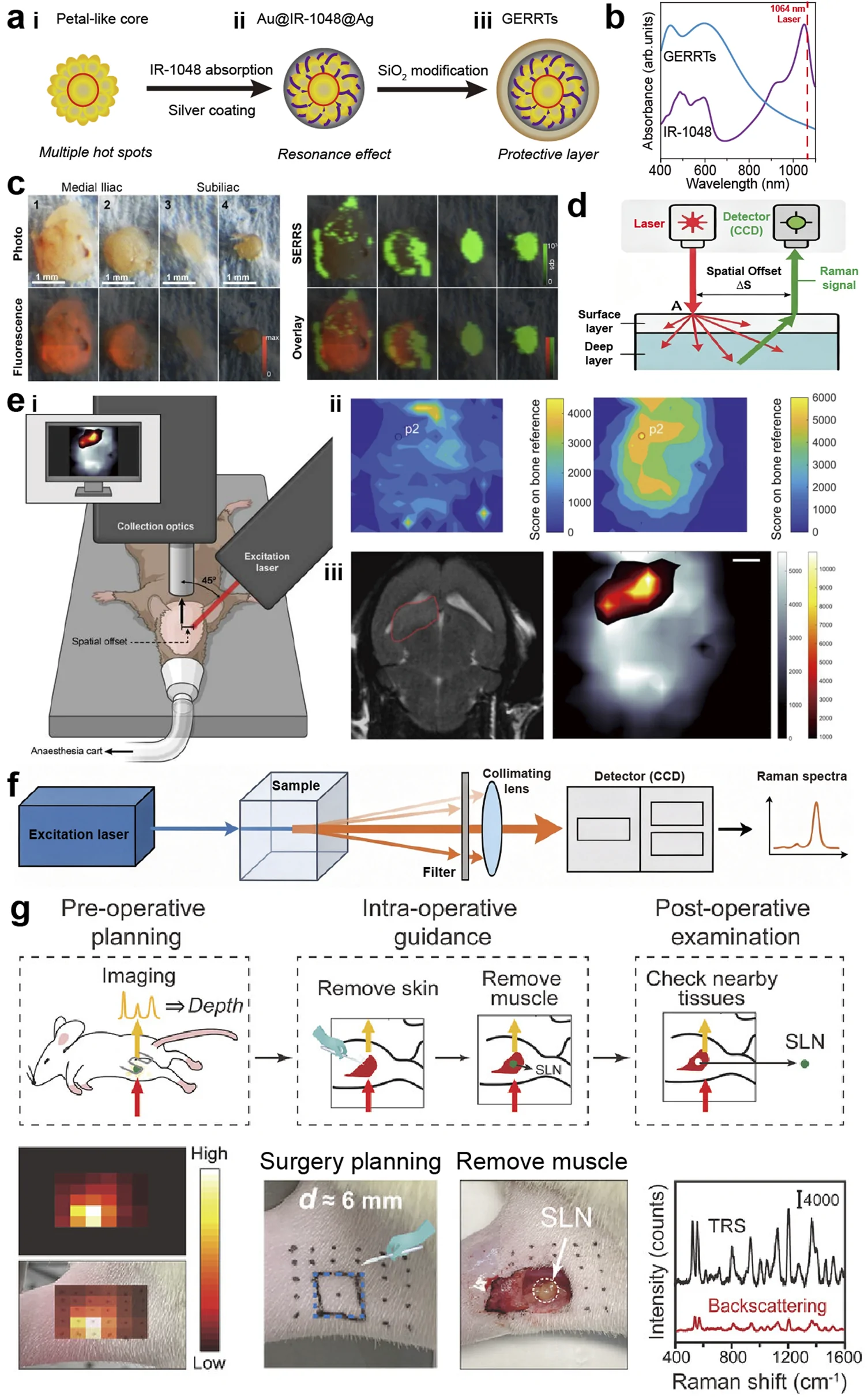
Principles and biomedical applications of advanced derivative Raman technologies. **(a)** Synthesis illustration of SERRS nanoparticles (Note: Original image). **(b)** Experimental extinction spectra of aqueous GERRTs and IR-1048 iodide. The dotted line indicates the laser wavelength (1064 nm) used in Raman measurements. (Note: Original image) **(c)** *Ex vivo* SERRS imaging for detecting metastatic infiltration in a prostate cancer mouse model (adapted with permission from Spaliviero et al., 2016, Copyright © 2016, World Molecular Imaging Society). **(d)** The SORS concept (Note: Original image). **(e)** *In vivo* SESORS imaging of glioblastoma multiforme (GBM) through the intact skull: (i) intravenous delivery of targeted SERRS nanoparticles in a murine GBM model; (ii) Comparison of bone signal detection using conventional Raman (CR, left) versus SESORS (right) approaches; (iii) T2-weighted MRI of the tumor (left) and the corresponding SESORS heatmap superimposed on the bone SORS image (right) (adapted from Nicolson et al. 2019, licensed under CC BY 4.0). **(f)** In TRS, the illumination and collection zones are on the opposite sides of the sample (Note: Original image). **(g)** *In vivo* SETRS strategy for depth localization and perioperative surgical navigation of an SLN in a rat model, showing pre-operative planning and intra-operative guidance to expose the node in situ (adapted from Wu et al. 2023, licensed under CC BY 3.0).

While conventional SERS provides substantial signal amplification, its sensitivity is often insufficient for detecting microscopic residual disease in complex *in vivo* environments. To overcome this limitation, surface-enhanced resonance Raman spectroscopy (SERRS) pushes the boundary of optical molecular imaging. When the excitation laser wavelength aligns simultaneously with the localized surface plasmon resonance of the substrate and the electronic transition absorption band of the Raman reporter, an overlapping effect of electromagnetic and chemical resonance enhancements occurs (Sreekanth et al., 2023). This dual-enhancement mechanism yields an additional 10^2^ to 10^3^ fold signal amplification relative to conventional SERS, propelling the total enhancement factor to 10^13^–10^15^ and enabling femtomolar or even attomolar limits of detection (Auner et al., 2018; Sun et al., 2019). To harness this extreme sensitivity, Zhang et al. developed gap-enhanced resonance Raman tags (GERRTs) (Zhang et al., 2023a). As illustrated in Figure 8a, the synthesis involves functionalizing a petal-like gold core with IR-780 iodide reporter molecules, followed by the encapsulation of an outer gold shell (Zhang et al., 2023a). Experimental extinction spectra (Figure 8b) reveal that the absorption profile of IR-780 aligns closely with the 785 nm excitation laser, effectively triggering the resonance Raman effect (Zhang et al., 2023a). Furthermore, finite-difference time-domain (FDTD) calculations confirm that this specific petal-like core-shell architecture generates intense electric field enhancement within the internal nanogaps. This precise integration of molecular resonance and structural electromagnetic amplification provides the ultrahigh sensitivity required for deep-tissue detection (Zhang et al., 2023a). Given the critical prognostic value of regional metastasis, SERRS has been extensively explored for LN assessment and surgical navigation. Spaliviero et al. investigated the detection of prostate cancer LN metastases using intravenously administered SERRS nanoparticles. As shown in Figure 8c, healthy LNs exhibited homogeneous SERRS signals due to avid nanoparticle uptake by resident macrophages; conversely, hypointense SERRS regions were detected within the metastatic LNs, consistent with the displacement of normal LN architecture by malignant infiltration (Spaliviero et al., 2016). Translating this to intraoperative navigation, Sun et al. used a handheld scanner to visualize metastatic LNs as hypo-intensive Raman regions based on SERRS-guided scanning (Sun et al., 2019). To expand deep-tissue capabilities, Li et al. developed NIR-II SERRS nanoprobes by conjugating bis(dithiolene)nickel dyes to gold nanorods, demonstrating exceptional brightness under 1064 nm irradiation (Li J. et al., 2023). Additionally, Iacono et al. achieved multiplexed *in vivo* visualization of distinct lymphatic drainage pathways using “schizophotonic” nanoparticles with different Raman reporters (Iacono et al., 2014). Finally, Wall et al. integrated preoperative PET-CT with intraoperative handheld SERRS tracking to precisely guide occult LN resection and confirm clean margins (Wall et al., 2017).

While SERRS offers extreme molecular sensitivity, conventional Raman spectroscopy remains restricted to superficial tissues. In highly turbid biological environments, intense photon scattering causes the collected signal to be almost entirely dominated by the surface layer. To overcome this depth limitation, Mosca et al. detailed the mechanism of spatially offset Raman spectroscopy (SORS) (Mosca et al., 2021). As illustrated in Figure 8d, SORS introduces a spatial displacement between the laser excitation point and the signal collection area (Mosca et al., 2021). This approach relies on the physical behavior of light: photons penetrating deeper into a diffusely scattering sample migrate laterally to a greater extent than those generated near the surface. By applying this spatial offset, the system bypasses the intense signals from unmigrated surface photons, selectively capturing laterally migrating, diffusely scattered photons that carry chemical information from deep-seated targets (Mosca et al., 2021). To realize this optical principle in hardware, the instrument design must physically separate the light source from the collection path. This rearranged excitation-collection geometry prevents the direct collection of superficially backscattered light. It provides the physical architecture necessary for depth-resolved diagnostics, making it possible to non-invasively probe deeply buried LNs without surgical exposure. To transition from experimental models to routine clinical practice, SORS technology must evolve into miniaturized optical probes. Keller et al. developed a handheld SORS probe specifically for intraoperative surgical margin assessment (Keller et al., 2011). By utilizing an array of detector rings, this probe captured spatially offset spectra to precisely discriminate between normal adipose tissue and invasive ductal carcinoma across a true tissue boundary (Keller et al., 2011).

Although SORS effectively retrieves chemical information from deep tissues, relying solely on endogenous Raman scattering often yields a low signal-to-noise ratio. When targeting highly occult and microscopic metastatic lesions, this weak endogenous signal is insufficient for precise diagnostic evaluations. To overcome this macroscopic-to-microscopic bottleneck, surface-enhanced spatially offset Raman spectroscopy (SESORS) was introduced. As illustrated in Figures 8e–i, SESORS represents a synergistic strategy: it utilizes SERS nanoprobes to provide ultra-bright, specific molecular signals, while employing the spatial offset collection mechanism of SORS to penetrate overlying healthy tissues and extract these signals. Nicolson et al. demonstrated the profound capability of SESORS in breaking the depth limit for *in vivo* imaging (Nicolson et al., 2019). Initially using a tissue phantom, they successfully generated 2D SORS heat maps of a target buried beneath 2 mm of tissue and an intact skull, applying spatial offsets ranging from 2 to 3 mm (Nicolson et al., 2019). Building upon this, they achieved a significant breakthrough in an *in vivo* glioblastoma mouse model. As shown in Figures 8e-ii, compared to conventional Raman imaging (left), SESORS (right) exhibits a signal intensity that is an order of magnitude higher, thereby successfully enabling high-contrast, non-invasive imaging of deep-seated lesions without the need for any surgical incisions. The technique accurately delineated the deep tumor margins through the intact skull, maintaining excellent spatial agreement with preoperative MRI and postoperative H&E histopathology (Figures 8e-iii) (Nicolson et al., 2019). To address the prolonged acquisition time of traditional point-by-point raster scanning in deep-tissue imaging, Nicolson et al. optimized data acquisition algorithms and sampling strategies for SESORS (Nicolson et al., 2024). By balancing sampling frequency with image resolution, their par-sampling approach successfully acquired a complete deep-tissue tumor image at a depth of 4.25 mm in just 325 s. This highly efficient scanning strategy significantly accelerates real-time intraoperative navigation without compromising diagnostic accuracy, as flawlessly validated by MRI and H&E results (Nicolson et al., 2024).

While SORS effectively extracts subsurface signals through lateral spatial offsets, clinical practice often requires a macroscopic evaluation of the entire internal volume when dealing with intact, thick tissues, such as excised LNs. According to the theoretical framework established by Mosca et al., transmission Raman spectroscopy (TRS) can be conceptually viewed as an extreme configuration of SORS (Mosca et al., 2021). As illustrated in Figure 8f, TRS positions the laser excitation and signal collection at exact opposite sides of the sample, effectively making the spatial offset equal to the total tissue thickness. This forward-scattering geometry forces photons to traverse the entire sample, endowing TRS with a unique “bulk volumetric sampling” capability. However, while conventional TRS can confirm the presence of internal targets, it inherently lacks depth-resolving power, failing to provide precise spatial coordinates for hidden lesions. To overcome this three-dimensional localization challenge and further push the limits of tissue penetration, researchers combined the transmission optical path with ultrabright SERS nanoprobes, bringing surface-enhanced transmission Raman spectroscopy (SETRS) into the realm of *in vivo* deep-tissue detection. Within the context of lymphatic mapping, identifying the precise depth of a node is a prerequisite for surgical intervention. To address this, Wu et al. developed an *in vivo* ratiometric SETRS strategy (Wu et al., 2023). By analyzing the intensity ratio of distinct Raman spectral peaks, this method accurately calculates subsurface depth. As illustrated in Figure 8g, this approach enabled the non-invasive localization and perioperative surgical navigation of a SLN in a rat model. By integrating the TRS image overlay with bright-field imaging, researchers conducted pre-operative surgical planning based on the depth prediction results, which successfully guided the precise removal of overlying muscle to expose the LN *in situ* (Wu et al., 2023).

Beyond regional LNs, SETRS demonstrates exceptional capabilities for detecting deep-seated solid tumors. Zhang et al. established a critical foundation for clinical viability by demonstrating non-invasive tumor detection under strict maximum permissible exposure safety limits (Zhang et al., 2023a). Their transmission system achieved photosafe *in vivo* detection of deep-buried tumor phantoms in mice, providing clear SERS overlap images through the tissue (Zhang et al., 2023a). Advancing toward true three-dimensional localization, Xie et al. introduced tomographic transmission Raman spectroscopy to navigate complex tumor masses (Xie et al., 2023). By performing systematic ring scanning within an axial tissue stack, the system successfully located multiple SERS phantom lesions across different tissue layers, matching their actual physical locations (Xie et al., 2023). Transitioning these optical capabilities into routine clinical practice requires overcoming complex physiological variables. Zhang et al. optimized ratiometric algorithms to ensure depth estimation remains reliable even within highly heterogeneous biological tissues (Zhang et al., 2023b). Their depth prediction model maintained a predictable relationship with depth through a 5 cm thick stacked tissue block, effectively overcoming complex anatomical scattering pathways (Zhang et al., 2023b).

## 5 Clinical translation of Raman spectroscopy in sentinel lymph node biopsy: challenges and prospects

### 5.1 Current clinical translation challenges

Despite significant advances in optical molecular imaging, translating these technologies from laboratory prototypes to routine SLNB faces multiple systemic hurdles. These constraints are often related to high costs, complex equipment maintenance, and a steep learning curve for surgeons (Li et al., 2026). A primary obstacle is the substantial gap between *ex vivo* models and the complex *in vivo* physiological environment (Wu et al., 2023). For instance, depth prediction models trained on frozen tissues perform poorly in living subjects, as the freezing process induces irreversible physicochemical alterations and structural damage (Zhou et al., 2024). When optical parameters derived from frozen tissues are applied to *in vivo* subjects, the relative standard deviation (RSD) of the predicted depth can exceed 50% (Zhou et al., 2024). Furthermore, executing non-invasive detection under the strict clinical maximum permissible exposure (MPE) for lasers is technically demanding (Wu et al., 2023). Within safe irradiance limits, conventional Raman signals often suffer from low signal-to-noise ratios and severe interference from tissue autofluorescence (Kim et al., 2023). Patient heterogeneity, including variations in age, race, and tissue composition, further destabilizes the reproducibility of spectral readings (Papadoliopoulou et al., 2023). Another core challenge lies in the nanoscale heterogeneity of SERS substrates. Fabricating uniform and stable distributions of plasmonic “hot spots” remains highly difficult, which directly undermines signal reproducibility and limits reliable quantitative analysis (Chen et al., 2025). Additionally, inter-laboratory comparisons are severely restricted by the absence of unified detection standards and quality control systems for sample preprocessing and signal acquisition (Ma M. et al., 2024; Plou et al., 2022). From a regulatory and clinical workflow perspective, the toxicity and long-term metabolic clearance of exogenous metal nanoparticles necessitate rigorous biosafety evaluations aligned with FDA and ISO standards before human application. Currently, most Raman systems are research-grade and lack the clinical robustness required for medical device approval by agencies such as the FDA or EMA (Bufka et al., 2025). Moreover, interpreting complex Raman spectra requires specialized spectroscopic expertise, which is laborious for clinicians and disrupts standard surgical workflows (Ma M. et al., 2024). Ultimately, comprehensive health economic evaluations are missing, yet they are mandatory for incorporating these emerging technologies into medical insurance payment strategies (Luo et al., 2025).

### 5.2 Future development prospects

To navigate these translational barriers, the future of Raman-guided SLNB relies on interdisciplinary convergence. The integration of AI offers a powerful solution to data complexity, and automated spectral diagnostic frameworks must be developed to alleviate the cognitive burden on medical staff (Correia, 2025). A particularly promising advancement is the use of generative AI (GenAI) models to directly synthesize hematoxylin-eosin (H&E)-stained histopathological images from Raman mapping data (Yan et al., 2025). This “virtual pathology” approach transforms abstract spectral patterns into familiar histological features, enabling rapid, non-invasive margin assessment without disrupting the pathologist’s conventional visual workflow (Yan et al., 2025). However, to ensure clinical trust, future AI models must overcome their “black-box” nature and be validated on ethnically diverse, multi-center datasets to prevent overfitting (Bufka et al., 2025; Zhu et al., 2025). In terms of probe design, there is a strong trend toward all-in-one composite tracers that align with established surgical practices. Clinically, SLN mapping typically requires multiple sequential injections of radioactive colloids and blue dyes, imposing logistical burdens (Deng et al., 2025). Recent innovations have merged SERS nanotags with MB to create a single-injection dual-tracer (Deng et al., 2025). This strategy preserves the surgeon’s reliance on visual identification (blue staining) while providing high-precision molecular confirmation through Raman scanning (Deng et al., 2025). Additionally, utilizing donor-acceptor (D-A) conjugated polymers as pure organic Raman probes shows great potential for entirely circumventing intrinsic fluorescence background interference during *in vivo* imaging (Bao et al., 2024). Finally, the hardware evolution toward cost-effective, commercial portable Raman scanners is accelerating clinical feasibility (Deng et al., 2024b). These miniaturized devices facilitate on-site, real-time measurements in resource-limited surgical settings. In the future, large-scale, multicenter clinical trials evaluating both diagnostic accuracy and health economics will be the decisive step in establishing RS as a routine clinical standard (Bufka et al., 2025; Luo et al., 2025).

## 6 Conclusion

SLNB remains the gold standard for staging regional LN metastasis and guiding clinical treatment decisions for various solid tumors. However, conventional intraoperative detection methods, such as frozen section analysis and touch imprint cytology, face significant clinical limitations, including high subjectivity, long detection times, and low sensitivity for micrometastases. RS provides a compelling alternative as a label-free, non-destructive optical molecular imaging technology capable of obtaining the intrinsic biochemical fingerprint information of tissues. To overcome the inherent weaknesses of conventional RS, namely, limited penetration depth and weak signal intensity, derivative techniques, particularly SERS, have been developed to realize ultra-sensitive and targeted SLN detection.

While the application of RS, SERS, and their derivative technologies in SLNB for tumors such as breast cancer, thyroid cancer, and melanoma has shown immense preclinical promise, transitioning these technologies into routine clinical practice requires overcoming significant translational hurdles. Future development directions must address these clinical translation challenges through interdisciplinary efforts to optimize nanotracers, ensure biosafety, standardize detection protocols, and seamlessly integrate optical devices into surgical workflows. Ultimately, the continued evolution of RS technologies aims to deliver a rapid, accurate, and objective intraoperative SLN detection platform, serving as a vital reference for the future of precision oncology and individualized patient care.
